# Modulating the PCET process *via* optimizing the local microenvironment of a CdS@NiV-LDH heterojunction for CO_2_ reduction in tunable green syngas photosynthesis†

**DOI:** 10.1039/d4sc07856j

**Published:** 2025-02-10

**Authors:** Senlin Zhang, Yuheng Ma, Changqiang Yu, Zhaohui Huang, Ruoning Zhan, Yingxinjie Wang, Xiuqiang Xie, Nan Zhang

**Affiliations:** a College of Materials Science and Engineering, Hunan University Changsha 410082 China xiuqiang_xie@hnu.edu.cn nanzhang@hnu.edu.cn

## Abstract

Photoconversion of CO_2_ and H_2_O into syngas (CO + H_2_) for the Fischer–Tropsch reaction is considered a feasible plan to address global energy requirements in times of global warming. However, the production of syngas with high activity and adjustable proportion is challenging mainly due to the less efficient multi-step proton-coupled electron transfer (PCET) process owing to the unfavorable local microenvironment of photocatalysts. Herein, an S-scheme CdS@NiV-LDH (HNV) heterojunction is constructed through mild wet-chemistry methods, and NiV-LDH nanosheets are uniformly grown *in situ* on the surface of hollow cubic CdS (HCC). The as-prepared three-dimensional hierarchical architecture of the HNV photocatalyst leads to a controllable CO/H_2_ ratio ranging from 0.2 to 1, and the CO and H_2_ production rate of the optimal HNV-4 heterojunction can reach 1163.8 μmol g^−1^ h^−1^ and 1334.6 μmol g^−1^ h^−1^, respectively. X-ray photoelectron spectroscopy, electron spin spectroscopy, and photo-deposition platinum metal test show that the photogenerated charge carriers in HNV follow an S-scheme charge transfer mechanism. This significantly improves the spatial separation of the photogenerated electron–hole pairs *via* the built-in electric field that modifies the electric field microenvironment of the HNV photocatalyst to accelerate the photoreduction process. Meanwhile, the NiV-LDH nanosheets on the external surface act as CO_2_ enricher and H_2_O moderator that adjusts the reaction microenvironment to speed up the PCET process by increasing the local CO_2_ concentration and facilitating *COOH intermediate generation in the HNV heterojunction. This work opens a new horizon for exploring novel heterogeneous photocatalysts toward enhanced visible-light-driven CO_2_ conversion to tunable green syngas.

## Abstract 2

A novel CdS@NiV-LDH heterojunction with an optimized electric field microenvironment and reaction microenvironment has been constructed for the photocatalytic reduction of CO_2_ to tunable green syngas by rationally modulating the PCET process.

## Graphical abstract

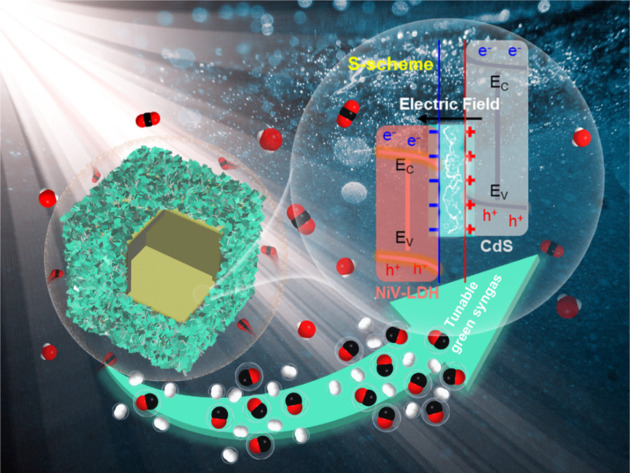

## 1. Introduction

In the last century, the increasing demand for energy in daily life, extensive reliance on traditional fossil fuels, and ongoing deforestation have led to a continuing increase in the atmospheric carbon dioxide (CO_2_) level.^1–3^ Inspired by the natural photosynthesis process, researchers have found that solar energy-mediated photocatalytic pathways hold promising and durable solutions to achieve the goals of “carbon neutrality” by efficiently converting CO_2_ into high-value-added chemicals and fuels.^4–6^ There are many types of CO_2_ photoreduction products (such as CO, HCOOH, CH_3_OH, CH_4_, C_2_, and C_2^+^_), of which carbon monoxide (CO) has attracted special attention as one of the most valuable raw materials for Fischer–Tropsch synthesis (FTS) to produce hydrocarbon liquid fuels, which is expected to replace petroleum-based processes.^7,8^ In industrial processes, adjusting the ratio of CO and hydrogen (H_2_) in syngas is essential to meet specific chemical requirements due to that the syngas (*i.e.*, a mixture of CO and H_2_) is a versatile feedstock for FTS.^9^ For instance, dimethyl ether, methanol, and syngas fermentation can be used to give products with ratios of CO/H_2_ at 0.3, 0.5, and 1, respectively. Syngas is predominantly produced in harsh synthetic conditions and proportionally uncontrollable, which is traditionally obtained by coal gasification, natural gas reforming, or water–gas reforming reactions.^10^ Photocatalytic CO_2_ reduction to syngas can be achieved at room temperature (*ca.* 25 °C) and normal pressure (1 atm) using solar energy alone.^11^ However, the efficiency of current photocatalytic CO_2_-to-syngas reduction systems is still unsatisfactory, mainly due to the easy recombination of charge carriers, insufficient activation of CO_2_ molecules, and competing hydrogenation reaction.^12,13^ These factors restrict the reaction kinetics seriously and lead to unfavorable syngas ratios.^14^ Therefore, developing photocatalytic materials enabling efficient charge separation, outstanding CO_2_ activation, and widely adjustable syngas ratio is considered an available strategy to improve CO_2_ reduction efficiency, but remains challenging.

Among the semiconductor materials reported for CO_2_ photoreduction, cadmium sulfide (CdS) has been a focus of research due to its narrow band gap (*ca.* 2.4 eV), adequate negative conduction band potential for the reduction of CO_2_ to CO, and nanosize-tunable electronic structure.^15,16^ Moreover, hollow-structured photocatalysts with large surface areas and abundant active sites show distinct advantages for solar energy conversion reactions.^17^ For instance, the thin shells of a hollow structure could reduce the distance for the transfer of photogenerated charge carriers, and the light scattering effect can be enhanced, which would lead to enhanced light absorption capability.^18–20^ However, the photocatalytic efficiency of CO_2_-to-syngas conversion over conventional CdS-based catalysts is still low, accompanied by an inapplicable syngas ratio with a too great a share of H_2_ compared to CO.^21,22^ This is mainly caused by the inherent microenvironment of CdS with a weak electric field microenvironment leading to inefficient photogenerated carrier separation capability,^23^ and an unsuitable reaction microenvironment leading to inadequate CO_2_ adsorption and activation and thus unsatisfactory product distribution.^24,25^ Meanwhile, the class of material of layered double hydroxides (LDHs) with alkalinity has attracted great attention in the field of photocatalytic CO_2_ reduction due to their favorable CO_2_ adsorption capability.^26–28^ Tanaka *et al.*^29^ for the first time demonstrated the photocatalytic conversion of CO_2_ (dissolved in water) into CO and O_2_ on various M^2+^–M^3+^ LDHs. More recently, NiAl-LDH was developed for photocatalytic CO_2_ reduction, with a CO evolution rate of 1.01 μmol g^−1^ h^−1^, and the selectivity towards CO attaining almost 59.8%.^30^ Although native LDHs have exhibited great potential for photocatalytic reduction of CO_2_ to CO, the drawbacks, especially the sacrificing of catalytic active sites on LDHs due to the aggregation effect during the synthesis process, still limit the conversion efficiency.^31^

Recent research has revealed that the overall CO_2_ photoreduction with H_2_O is a proton-coupled electron transfer (PCET)-involved complex surface–interface reaction process, which is controlled by the intricate microenvironment provided by unique photocatalysts.^32,33^ It is worth noting that the thermodynamic and kinetic processes of protons (H^+^) produced from H_2_O molecules in the presence of both CO_2_ and H_2_O are sophisticated.^24,25,31^ Unlike the reaction system that aims to realize efficient CO_2_ reduction and simultaneously minimize proton coupling to H_2_ production,^21,34^ the reduction of CO_2_ and H_2_O to green syngas needs to “balance” the CO_2_ reduction and proton reduction processes. In this situation, it is essential to unveil whether the protons prefer to bind with CO_2_˙^−^ to generate *COOH intermediate for efficient generation of the product CO through the PCET process or rapidly to couple to each other to produce H_2_. More importantly, revealing how microenvironment engineering affects the above processes is of significant importance to obtain green syngas with tunable CO/H_2_ ratio from photoreduction of CO_2_ and H_2_O. However, the research in this regard is still scarce.^35^

Herein, an S-scheme heterojunction CdS@NiV-LDH (HNV) photocatalyst is constructed by mild wet-chemistry methods and applied to reduce CO_2_ for syngas synthesis. The HNV heterojunctions are adjusted by varying the content of NiV-LDH to achieve the photocatalytic reduction of CO_2_ into syngas with adjustable ratios in the wide range of 0.2–1. The total generation rate of green syngas from photoreduction of CO_2_ by the optimal HNV is 2498.4 μmol g^−1^ h^−1^. Various complementary characterizations collectively confirm that the special three-dimensional (3D) hierarchical architecture of the optimal catalyst with hollow CdS nanocube inner cavity and rough external surface modified by NiV-LDH nanosheets has efficient visible light absorption, rapid charge separation and migration, and favorable localized CO_2_ concentration and mass transfer efficiency. Based on the above, this well-designed photocatalyst with an optimized electric field microenvironment and reaction microenvironment can significantly increase syngas yield as well as achieve tunable CO/H_2_ ratios by accelerating the PCET process to facilitate the formation of *COOH intermediate. The special 3D hierarchical architecture may shed light on the rational design of advanced materials and reaction systems for photocatalytic CO_2_ reduction to produce green syngas.

## 2. Experimental section

### 2.1 Materials

All materials were of analytical grade and used as received without further purification. Detailed information is provided in the ESI.†

### 2.2 Preparation of photocatalysts

Experimental procedures for preparing different photocatalysts in this study are provided in the ESI.†

### 2.3 Characterization

Specific details are provided in the ESI.†

### 2.4 Photoelectrochemical measurements

Photoelectrochemical measurements were carried out using an electrochemical workstation (CHI 660E, Shanghai Chenhua, China) with a conventional three-electrode system. Specific operational information is provided in the ESI.†

### 2.5 Measurement of photocatalytic CO_2_ reduction

Reactions of photocatalytic CO_2_ reduction were conducted in a custom-made reactor with a 300 W Xe lamp with a 420 nm cutoff filter (PLS-SXE 300E, Beijing Perfect Light Co. Ltd) and analyzed using a gas chromatograph (Shimadzu GC-2014, 5 Å molecular sieve column). Specific operational information is provided in the ESI.†

### 2.6 Calculation of apparent quantum yield of syngas production

Monochromatic lights with wavelengths of 365, 450, 500, and 605 nm were used for irradiation for 1 h. Specific operational information is provided in the ESI.†

### 2.7 Experiment of *in situ* photodeposition

H_2_PtCl_6_·H_2_O solution containing 3 wt% Pt relative to the photocatalyst was added dropwise to a suspension of the HNV-4 photocatalyst. After degassing and backfilling the mixture with N_2_, the suspension was then irradiated for 1 h with a 300 W Xe lamp (*λ* > 420 nm). Specific operational information is provided in the ESI.†

## 3. Results and discussion

**Fig. 1:**
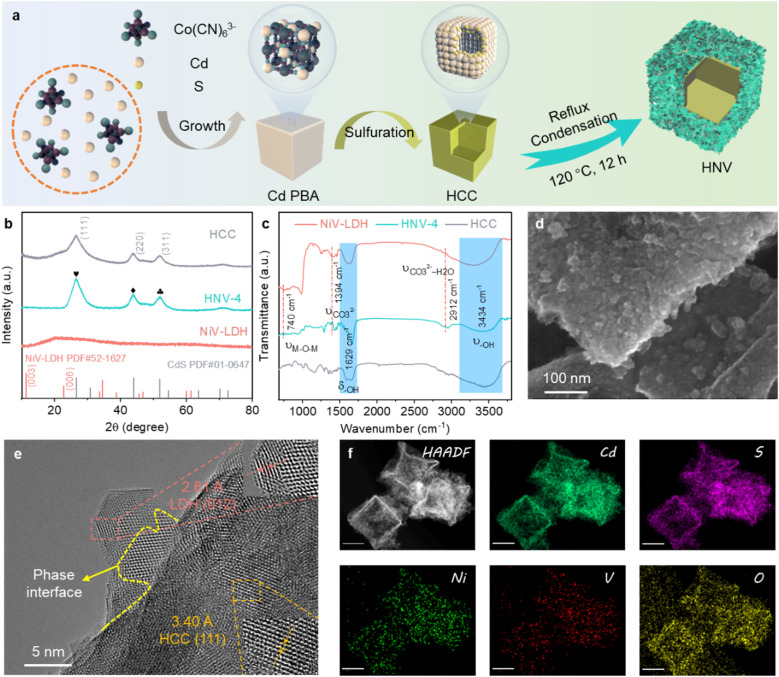
(a) Schematic flowchart for synthesis of HNV composite. (b) XRD and (c) FT-IR patterns of HCC, NiV-LDH, and HNV-4. (d) SEM and (e) HR-TEM images of HNV-4. (f) HAADF-STEM and corresponding EDS element mapping images of HNV-4 (scale bar: 200 nm).

The fabrication process of the CdS@NiV-LDH (HNV) composite is displayed in Fig. 1a. Cadmium Prussian blue analog (Cd PBA) precursor is synthesized by a facile solution-based ion exchange/precipitation method (the ion exchange of K^+^ to Cd^+^). Then, Cd PBA is converted into hollow cadmium sulfide cubes (HCC) through a sulfidation process using TAA and Na_2_S as sulfur sources.^36^ Finally, NiV-layered double hydroxide (NiV-LDH) nanosheets (NSs) are grown *in situ* on the surface of HCC through the one-pot reflux method. The as-prepared composite samples with different loadings of NiV-LDH NSs are named HNV-*X* (*X* = 1, 2, 3, 4, 5 corresponding to increasing content of NiV-LDH) (Table S1†). The crystalline structure of the samples is investigated using X-ray diffraction (XRD), the results of which are displayed in Fig. 1b and S1.† For the pure HCC, the distinct diffraction peaks at 26.4°, 43.8°, and 51.8° are indexed to the (111), (220), and (311) crystal planes of cubic hawleyite cadmium sulfide (JCPDS no. 01-0647), respectively.^18^ The XRD pattern of NiV-LDH exhibits a wide diffraction peak appearing at *ca.* 20.3°, corresponding to the (006) crystal plane of NiV-LDH (JCPDS no. 52-1627), and the peak for NiV-LDH NSs is shifted to a lower angle *ca.* 2.5° compared to the standard card, which might be ascribed to the enlarged interlayer space of the (006) plane or the formation of monolayer NiV-LDH NSs.^37^ In addition, the diffraction peak at 11.6° corresponding to the (003) crystal plane of NiV-LDH cannot be observed, which belongs to the characteristic peak of layered structure for LDHs, indicating that the as-prepared NiV-LDH does not have an obvious multilayer structure.^38^ The diffraction pattern of NiV-LDH cannot be observed for HNV composites (Fig. S1†). To understand this phenomenon, the accurate content of NiV-LDH in the composite is determined (Table S2†). Taking HNV-4 as an example, the NiV-LDH loading content is 48.0 wt%, which value should reach XRD detectability. According to the above analysis, the absence of the NiV-LDH XRD peaks in the pattern of the HNV composites should be ascribed to the large interlayer space and monolayer structure of NiV-LDH as well as the uniform distribution of NiV-LDH nanosheets on the surface of HCC. There are no obvious changes in characteristic peaks of HCC for the composite samples, indicating that the crystal structure of cubic hawleyite cadmium sulfide (CdS) does not change in the formation process of HNV with the introduction of NiV-LDH.^39^ Fourier transform infrared (FT-IR) spectroscopy is applied to investigate the chemical environment of samples. Fig. 1c displays the FT-IR spectra of HCC, NiV-LDH, and HNV-4, all of which exhibit broad and strong absorption peaks at 3434 cm^−1^ and 1629 cm^−1^, indicating the presence of stretching and bending modes of hydroxyl (–OH) groups, arising from adsorbed water molecules and metal-hydroxyl groups.^40^ Interlayer anions CO_3_^2−^ and CO_3_^2−^–H_2_O of NiV-LDH are confirmed through vibration peaks at 1394 cm^−1^ and 2912 cm^−1^, respectively, confirming the presence of hydrogen-bonded water molecules with carbonate anions in the interlayer of NiV-LDH.^28^ The absorption peak at 740 cm^−1^ (less than 800 cm^−1^) is ascribed to the stretching vibrations of metal–oxygen bonds present in NiV-LDH.^41^ In comparison with HCC and NiV-LDH, all FT-IR characteristic peaks of the HNV-4 composite are consistent with that of the pure component, indicating that the NiV-LDHs are successfully combined with HCC.

The morphology and microstructure of the as-prepared samples are investigated using scanning electron microscopy (SEM) and high-resolution transmission electron microscopy (HR-TEM). After the sulfidation reaction of the Cd PBA template, HCC is formed through a mechanism similar to the nanoscale Kirkendall effect,^42^ and the surface of HCC is smooth and uniformly dispersed (Fig. S2a†). Moreover, there is a slight collapse on the geometric surfaces of HCC, which is caused by the inward shrinkage of the nano-cubic nuclei during the sulfidation process.^18^ It can be seen from Fig. S2b† that pure NiV-LDHs demonstrate smooth nanosheet morphology with a size of 100–200 nm that agglomerate with each other. Compared to the single components, the surface of HNV-4 becomes rough (Fig. S2c†), and NiV-LDH NSs are uniformly distributed over the surface of the CdS nanocubes (Fig. 1d), which prevents the nanosheet from aggregating and provides more surface area and active sites for the rapid transport and migration of matter. As seen in the HR-TEM images (Fig. 1e and S3†), the lattice fringes of 3.40 Å and 2.61 Å are determined, which correspond to the (111) crystal facet of cubic CdS and the (012) crystal facet of NiV-LDH, respectively. Moreover, there is a clear interface intersection region between the two components, and the formation of tight contact interfaces plays a significant role in space separation and migration of electron–hole pairs. In addition, the uniform distribution of Cd, S, Ni, V, and O elements measured by energy dispersive spectrometry (EDS) further demonstrates the successful preparation of the three-dimensional (3D) hollow hierarchical CdS@NiV-LDH composite (Fig. 1f).

**Fig. 2:**
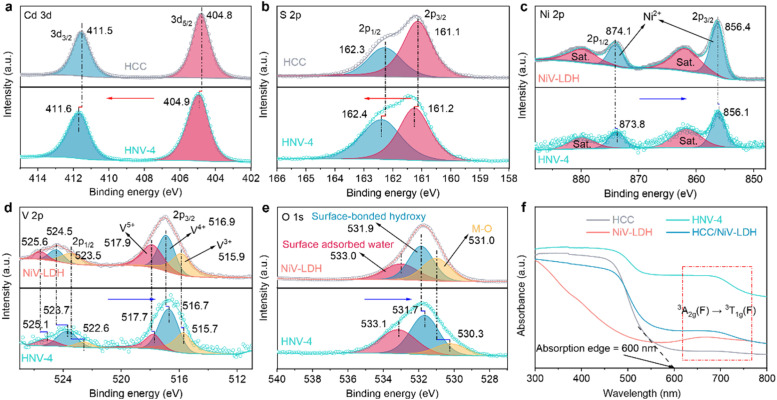
High-resolution XPS spectra of (a) Cd 3d and (b) S 2p of HCC and HNV-4 and (c) Ni 2p, (d) V 2p and (e) O 1s of NiV-LDH and HNV-4. (f) UV-visible diffuse reflectance spectra (DRS) of HCC, NiV-LDH, HNV-4, and HCC/NiV-LDH.

The surface elemental composition and chemical states of the samples are analyzed using X-ray photoelectron spectroscopy (XPS). Characteristic peaks of Cd, S, Ni, V, and O elements are detected in the HNV-4 composite (Fig. S4†), which is consistent with the high-angle annular dark-field scanning transmission electron microscopy (HAADF-STEM) element mapping results (Fig. 1f). As revealed in Fig. 2a, in the high-resolution Cd 3d spectrum of the HNV-4 composite, peaks situated at 404.9 eV (Cd 3d_5/2_) and 411.6 eV (Cd 3d_3/2_) are attributed to Cd^2+^ species. The high-resolution S 2p spectrum of the HNV-4 composite exhibits two featured peaks at 161.2 eV (S 2p_3/2_) and 162.4 eV (S 2p_1/2_), ascribed to S^2−^ species (Fig. 2b). Notably, compared with pristine HCC, the Cd 3d and S 2p peaks of the HNV-4 composite are shifted to higher binding energy, resulting from the decreasing electron cloud density of HCC in the HNV-4 composite.^22^ The high-resolution Ni 2p spectrum of HNV-4 shows two distinct peaks at 856.1 and 873.8 eV that correspond to Ni 2p_3/2_ and Ni 2p_1/2_, respectively, and two satellite peaks are also observed at 861.7 and 879.4 eV, indicating the native characteristic of Ni^2+^ spectra (Fig. 2c). As shown in Fig. 2d, the V 2p spectrum displays the presence of V 2p_3/2_ and V 2p_1/2_ peaks due to the spinning p orbital splitting. The high-resolution V 2p_3/2_ peak of HNV-4 can be deconvoluted into three binding energy components, corresponding to V^3+^ (515.7 eV), V^4+^ (516.7 eV), and V^5+^ (517.7 eV). The high-resolution V 2p_1/2_ peak of HNV-4 also can be deconvoluted into three binding energy components, corresponding to V^3+^ (522.6 eV), V^4+^ (523.7 eV), and V^5+^ (525.1 eV), implying that some V^3+^ was oxidized to V^4+^ and V^5+^ during the oil bath process.^40^ The high-resolution O 1s spectrum of HNV-4 can be deconvoluted into three distinct peaks located at 530.3, 531.7, and 533.1 eV that correspond to metal–oxygen (M–O, where M represents Ni or V) bonds, surface-bonded hydroxyl groups (–OH), and surface-adsorbed water molecules, respectively (Fig. 2e).^43^ Different from the peak value shifts of Cd 3d and S 2p, the core horizontal spectra of Ni 2p, V 2p, and O 1s for the HNV-4 composite shift to lower deconvolution peak values compared with pure NiV-LDH, resulting from the increasing electron cloud density of NiV-LDH in HNV-4, implying a charge accumulation on the surface of NiV-LDH in the composite. Compared to pure HCC and NiV-LDH, the relative shift direction of the main peaks for corresponding components in the HNV-4 composite suggests a strong interfacial interaction between HCC and NiV-LDH. In other words, the CdS@NiV-LDH heterostructure has been successfully prepared.

Based on the strong interaction between HCC and NiV-LDH, the light absorption capability of the HNV heterostructure exhibits distinct synergistic effects. As shown in Fig. 2f, HCC displays an obvious visible light response with an absorption edge extended to 600 nm, which is attributed to the intrinsic band gap photoexcitation of CdS. Pristine NiV-LDH possesses a wider light response range but lower intensity, with apparent light absorption across the range of wavelengths measured (200–800 nm). Resulting from spin–orbit coupling, the absorption band at 645–710 nm of NiV-LDH corresponds to the ^3^A_2g_(F) → ^3^T_1g_(F) transition of Ni^2+^.^38^ According to the Tauc plots (Fig. S5†), the corresponding bandgap (*E*_g_) of HCC and NiV-LDH is determined to be 2.40 and 1.73 eV, respectively. As for HNV heterostructures, both absorptions corresponding to HCC and NiV-LDH can be observed (Fig. S6†), indicating the successful combination of HCC and NiV-LDH. Notably, all of the heterostructures show a stronger absorption at 600–800 nm, and HNV-4 exhibits stronger absorption than bare HCC and NiV-LDH. In addition, the HCC/NiV-LDH composite prepared by mechanical milling exhibits significantly lower light-harvesting capacity than HNV-4, manifesting a more favorable synergistic effect in the CdS@NiV-LDH heterostructure that is constructed by an *in situ* growth strategy.

**Fig. 3:**
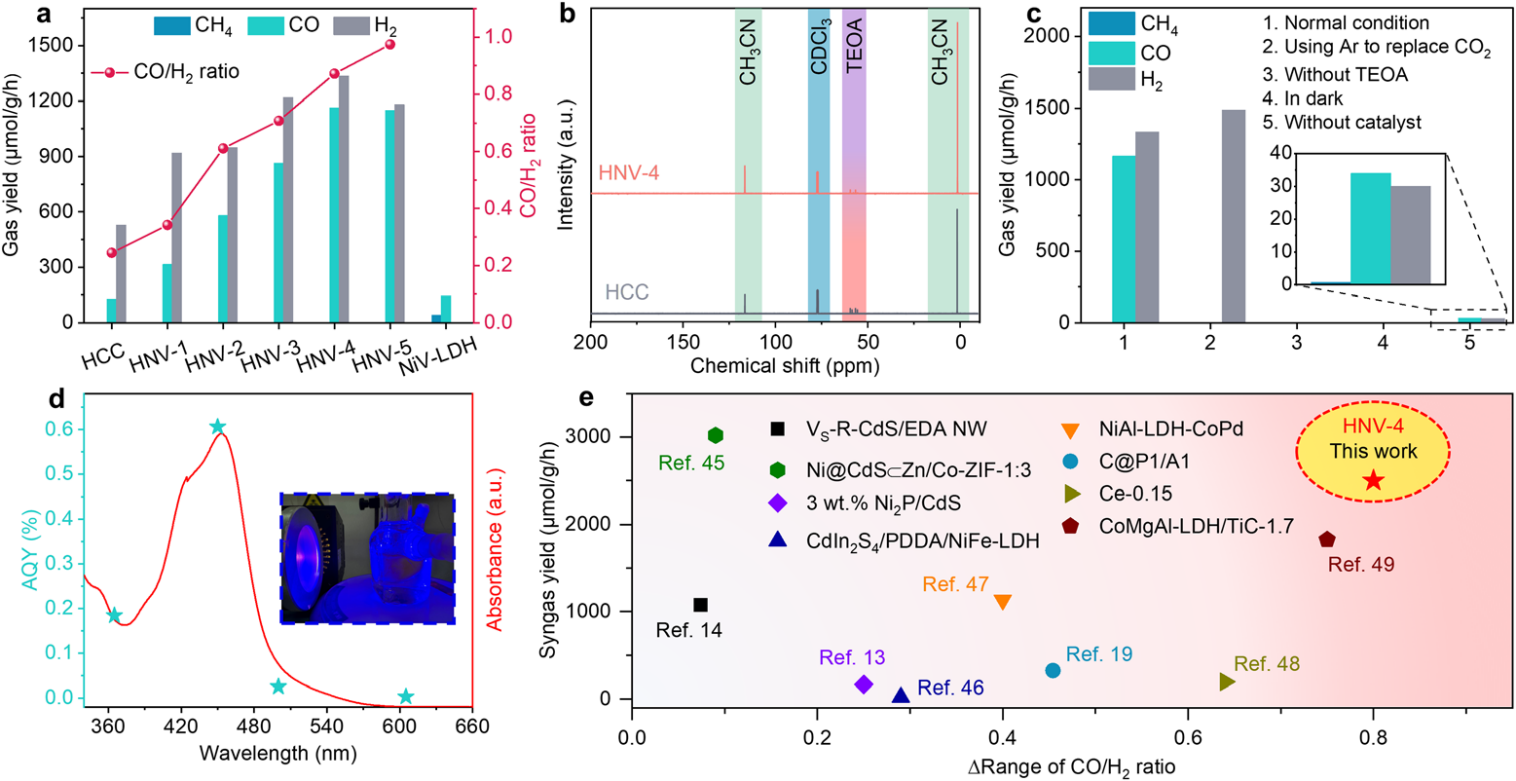
(a) Product yield over HCC, NiV-LDH, and HNV-*X* (*X* = 1, 2, 3, 4, 5). (b) ^13^C NMR spectra of liquid-phase products after photocatalytic CO_2_ reduction over HCC and HNV-4. (c) CO_2_ photoreduction of control experiment under different conditions. (d) AQY results of HNV-4 along with the absorption spectrum of the [Ru(bpy)_3_]Cl_2_·6H_2_O photosensitizer. The inset is a photograph of the corresponding experimental setup. (e) Tunable range of CO/H_2_ ratios and syngas yield of photocatalytic CO_2_ reduction over HNV-4 in comparison with some typical CdS-based and LDH-based photocatalysts.

Subsequently, the CO_2_ photocatalytic reduction (CO_2_PR) performance of the samples was studied under visible light irradiation (*λ* > 420 nm) in a mixture of acetonitrile (CH_3_CN) and water with triethanolamine (TEOA) as a sacrificial agent of photogenerated holes and [Ru(bpy)_3_]Cl_2_·6H_2_O as a photosensitizer. The gaseous and liquid products were analyzed using gas chromatography and nuclear magnetic resonance (NMR), respectively. As depicted in Fig. 3a, the CH_4_ and CO production rates over pure NiV-LDH are 41.2 μmol g^−1^ h^−1^ and 144.5 μmol g^−1^ h^−1^, respectively, and no other gas product such as H_2_ is observed. Products CO and H_2_ are mainly observed for HCC and HNV-*X* (*X* = 1, 2, 3, 4, 5), and the yield of CH_4_ is less than 2 μmol g^−1^ h^−1^. With increasing NiV-LDH NSs loading, the generation rates of CO and H_2_ gradually increase for HNV-*X* heterostructures, accompanied by an increase in the rate of syngas production. The optimal CO, H_2_, and syngas production rates are observed over HNV-4, which are determined to be 1163.8 μmol g^−1^ h^−1^, 1334.6 μmol g^−1^ h^−1^, and 2498.4 μmol g^−1^ h^−1^, respectively. The CO production rate of the optimal HNV-4 sample is approximately 9-fold and 8-fold that of pure HCC and NiV-LDH, respectively. Intriguingly, the ratio of the CO/H_2_ product over HNV-*X* heterojunctions can be tunable from 0.2 to 1, and the tunable syngas is not only an ideal feedstock for the Fischer–Tropsch process but also favors the synthesis of low-carbon olefins (C_2_–C_4_).^44^ Moreover, the liquid products have been detected by ^13^C NMR measurement after photocatalytic CO_2_ reduction over HCC and HNV-4. The signals in Fig. 3b are attributed to CH_3_CN, TEOA, and deuterated chloroform (CDCl_3_), respectively. No additional peaks appeared in the ^13^C NMR spectra other than those of the reaction solution and the test reagent, proving that no liquid products are generated in the present reaction system. By comparing the intensity of ^13^C NMR signals for HCC and HNV-4, it is noticed that the TEOA peak for HNV-4 is smaller than the corresponding peak intensity for HCC, which is attributed to the fact that the HNV-4 heterostructure effectively promotes the spatial separation of photogenerated electrons and holes, and more TEOA is consumed by the holes.

In the process of photocatalytic reduction of CO_2_, it is necessary to explore the impact of some major experimental conditions on the reduction products.^45^ When replacing CO_2_ with Ar as the gas source or in the absence of TEOA or light irradiation in the catalytic system, CO cannot be detected, indicating that CO_2_, sacrificial agent, and light irradiation are essential for the present catalytic reactions (Fig. 3c, groups 2, 3, 4). Furthermore, only small amounts of CO and H_2_ can be detected without the catalyst, because the photosensitizer could act as a molecular catalyst to form homogeneous catalytic systems (Fig. 3c, group 5).^13^ Compared with the high yield of the system under normal conditions, the trace amounts of CO and H_2_ generated using [Ru(bpy)_3_]Cl_2_·6H_2_O contribute barely to the catalytic performance, suggesting that the obtained green syngas mainly produced from the photocatalytic reduction over HNV-4. The variation trend of the apparent quantum yield (AQY) for different incident light wavelengths matches with the absorption spectrum of [Ru(bpy)_3_]Cl_2_·6H_2_O (Fig. 3d), and the highest AQY can be up to 0.606% at a monochromic wavelength of 450 nm (Table S3†). Meanwhile, these results indicate that our photoreduction CO_2_ system is driven by the photoexcitation of [Ru(bpy)_3_]Cl_2_·6H_2_O, generating electrons to achieve the reduction process, wherein the HNV-4 composite serves as a medium to accelerate electron transmission to reduce CO_2_. Furthermore, in the absence of [Ru(bpy)_3_]Cl_2_·6H_2_O, HNV-4 can reach an AQY of 0.162% at a monochromic wavelength of 365 nm, and the trend of the AQY with incident light wavelength matches with the UV-visible DRS of HNV-4 (Fig. S7 and Table S3†). As shown in Fig. 3e and Table S4†, the tunable range of CO/H_2_ ratio (*Δ* range of CO/H_2_ ratio) reaches 0.8, which is significantly higher than that of some typical CdS-based and LDH-based photocatalysts, while maintaining a high syngas yield.^15,16,22,46–50^ Besides, the HNV-4 sample after the photoreduction reaction was analyzed. It can be seen from the XPS spectra (Fig. S8†) that the chemical composition and the binding energy values of the elements for the used HNV-4 remain unchanged, indicating its favorable photostability.

**Fig. 4:**
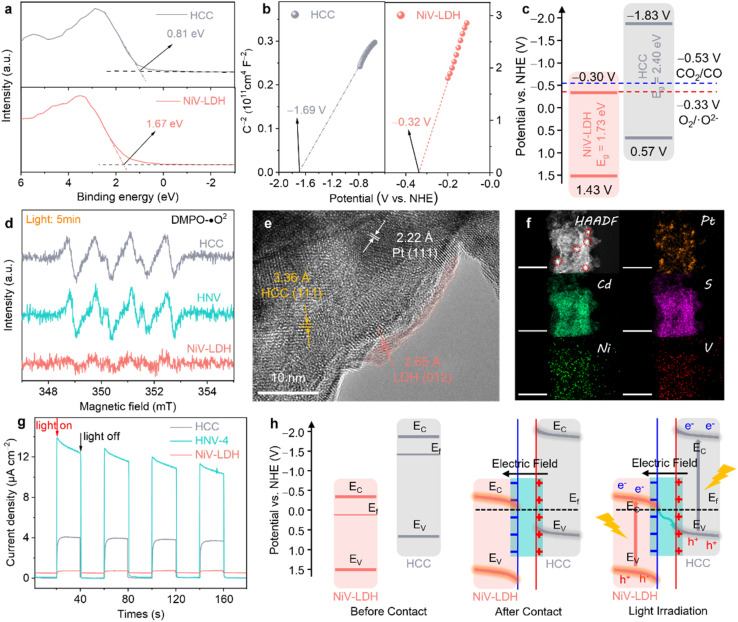
(a) XPS valence band spectra of HCC and NiV-LDH. (b) Mott–Schottky plots of HCC and NiV-LDH at 1000 Hz. (c) Band structure of HCC and NiV-LDH (*vs.* NHE). (d) ESR spectra of DMPO-˙O^2−^ adducts over HCC, NiV-LDH, and HNV-4 after irradiation. (e) HR-TEM and (f) HAADF-STEM and corresponding EDS element mapping images of HNV-4@Pt (scale bar: 200 nm). (g) Transient photocurrent responses of HCC, NiV-LDH, and HNV-4. (h) Proposed intrinsic mechanism of S-scheme electron migration pathway for HNV-4 heterojunction.

To reveal the underlying mechanism of the photocatalytic reduction process for the prepared catalysts, various complementary characterizations were conducted. The valence band (VB) of the samples was evaluated from the corresponding VB XPS spectrum (see Fig. 4a), where the corresponding *E*_VB,XPS_ of HCC and NiV-LDH are measured to be 0.81 and 1.67 eV, respectively. Then, *E*_VB_ of the corresponding standard hydrogen electrode (*E*_VB,NHE_) can be calculated according to the following formula: *E*_VB,NHE_ = *ϕ* + *E*_VB,XPS_ − 4.44, where *ϕ* is the work function of the instrument (4.20 eV). Thus, *E*_VB,NHE_ of HCC and NiV-LDH are calculated to be 0.57 and 1.43 V, respectively. Following the equation of *E*_FB,NHE_ = *E*_Ag/AgCl_ + *E*^*ϕ*^_Ag/AgCl_, where *E*^*ϕ*^_Ag/AgCl_ is 0.198 V, the flat band (FB) potentials of HCC and NiV-LDH are calculated to be −0.32 and −1.69 V *vs.* NHE by Mott–Schottky measurement, respectively (Fig. 4b). The positive slope of the fitting curve indicates that HCC and NiV-LDH are n-type semiconductors, and the conduction band value (*E*_CB_) for n-type semiconductors is usually more negative than *E*_FB_ of approximately 0.1–0.3 V.^30^ Moreover, according to the values of *E*_g_ and *E*_VB,NHE_, the values of *E*_CB,NHE_ for HCC and NiV-LDH are −1.83 and −0.30 V, respectively.^21^ As shown in Fig. 4c, if the photoinduced charge carriers are transported through a conventional type-II heterojunction route, the photogenerated electrons of HCC would migrate to the CB of NiV-LDH to reduce CO_2_ or protons (H^+^),^51^ while the CB potential of NiV-LDH is lower than the reduction potential of H^+^/H_2_ (−0.41 V *vs.* NHE) and CO_2_/CO (−0.53 V *vs.* NHE), indicating that the component of NiV-LDH cannot produce syngas from thermodynamics. Moreover, NiV-LDH can catalyze the production of CH_4_ (CO_2_/CH_4_, −0.24 V *vs.* NHE), in agreement with the results of Fig. 3a, and yet the products of HNV-*X* composite catalysis contain barely any CH_4_. Therefore, the type-II heterojunction mechanism is not suitable for the HNV-4 heterostructure.

The realistic charge transfer mechanism of the HNV heterostructure is validated through electron spin resonance (ESR) measurements. In detail, 5,5-dimethyl-1-pyrroline-*N*-oxide (DMPO) is used as a spin-trapping reagent in the HNV-4 heterostructure system, and ˙O^2−^ is generated by the electron injection to dissolved O_2_ in methanol, and the transient radical will transform to DMPO-˙O^2−^ adduct which is a stable radical that is suitable for ESR detection. As depicted in Fig. 4d, the six characteristic peaks assigned to DMPO-˙O^2−^ adducts are present for HCC and HNV-4 after 5 min of visible light irradiation, while no signal is observed for pure NiV-LDH, suggesting that photogenerated electrons used in the reduction reaction are mainly accumulated in the CB of HCC, not of NiV-LDH, whose *E*_CB_ is not negative enough to produce O_2_/˙O^2−^ (−0.33 V *vs.* NHE).^52^

To further prove the successful matching of S-scheme heterojunction, we then ascertained the directional charge flow in the HNV-4 heterostructure by site-specific platinum (Pt) photodeposition using the same light source that was used in the photocatalytic reduction of CO_2_. The as-prepared samples are denoted as HNV-4@Pt. Together with Fig. S9,† the HR-TEM image of HNV-4@Pt (Fig. 4e) shows lattice fringes of *ca.* 3.36, 2.65, and 2.22 Å, matching well with the (111), (012), and (111) crystal facets of HCC, NiV-LDH, and Pt nanoparticles (NPs), respectively.^53^ It can be observed that the Pt NPs tend to grow on the surface of HCC showing darker-colored black regions, and NiV-LDH exhibits small and thin nanosheets of light grey, suggesting that the photogenerated electrons tend to migrate to the surface of HCC for selective metal reduction. Generally speaking, the Pt NPs obtained by *in situ* photodeposition are 5–10 nm in size with uniform dispersion. As shown in Fig. 4f, the HAADF-STEM and corresponding EDS mapping images clearly show that many clear and large-sized (*ca.* 15–20 nm) bright Pt spots are more centrally distributed on the surface of the sample. According to the microstructure characteristics, NiV-LDH in the HNV-4 heterojunction uniformly covers the surface of HCC, resulting in a certain extent of masking of the active sites on HCC. As a result, a large number of photoelectrons accumulate on a small area of HCC and are then transferred to Pt^4+^, promoting the reduction of Pt^4+^ and continued growth and aggregation of Pt NPs.^54^ Assuming that Pt NPs are photodeposited on NiV-LDH, which is located in the outermost layer of the HNV-4 heterojunction, the size of the NPs should be relatively small and uniformly distributed. However, the above assumption is contrary to the experimental results in this work, which further indicate that the photogenerated electrons of HNV-4 are mainly accumulated on HCC. In other words, in the HNV-4 heterojunction, HCC acts as a reduction-type photocatalyst (RP) and NiV-LDH serves as an oxidation-type photocatalyst (OP).^55^

The interfacial charge separation and transfer efficiency of HCC, NiV-LDH, and HNV-4 were tested using photoelectrochemical measurements. As shown in Fig. 4g, the transient photocurrent response (TPR) intensity of the HNV-4 heterojunction is much higher than that of HCC and NiV-LDH, and the photoresponse curve of HNV-4 shows a spike at the moment the light source is turned on, owing to the instantaneous accumulation of light-induced charge carriers.^46^ Moreover, the Nyquist plot of HNV-4 in the electrochemical impedance spectra (EIS) shows the smallest radius under the cover of darkness, suggesting the lowest resistance to inner electron transport between HCC and NiV-LDH (Fig. S10†). These results are consistent with TPR testing, further confirming that the successful construction of the S-scheme heterojunction can effectively reduce the interfacial resistance of transferring photocarriers, which facilitates carrier migration and modifies the electric field microenvironment of the HNV heterojunction. According to the results of energy band structure, ESR, photodeposition of Pt NPs sites, and photoelectrochemical measurements, an S-scheme electron migration pathway is proposed (Fig. 4h). Due to the Fermi level (*E*_f_) of n-type semiconductors being near the CB, *E*_f_ of NiV-LDH is lower compared to that of HCC, and when NiV-LDH and HCC come into contact, the electrons inside HCC spontaneously migrate to NiV-LDH to reach an equilibrium of *E*_f_. Note that this is in good agreement with the results achieved for XPS in the ground state (Fig. 2a–e). Simultaneously, due to the enrichment of electrons on the surface of NiV-LDH, a built-in electric field (BIEF) from HCC to NiV-LDH is established, positively and negatively charged for HCC and NiV-LDH, respectively. With the presence of the BIEF inducing the occurrence of band bending of the semiconductor, the photogenerated electrons in the CB (−0.30 V *vs.* NHE) of NiV-LDH can slide downwards along the bent energy band to the VB (0.57 V *vs.* NHE) of HCC and recombine with holes, thus retaining a large amount of strong reducing electrons on HCC under visible light irradiation.^56^ The photogenerated electrons from the CB of HCC (RP) reduce CO_2_ and H_2_O to syngas, and the photogenerated holes from the VB of NiV-LDH (OP) are consumed by the sacrificial agent (TEOA). To sum up, the rational construction of the BIEF based on the S-scheme controllably modifies the electric field microenvironment of the HNV-4 heterojunction photocatalyst, which can promote the efficient separation of photoexcited charge carriers while maintaining high redox capabilities. However, it is non-selective that the kinetic support provided for the reduction of CO_2_ to CO associated with the PCET reaction and the splitting of H_2_O to H_2_ through the proton (H^+^) coupling process under the modulation of the electric field microenvironment alone.^24,57^

**Fig. 5:**
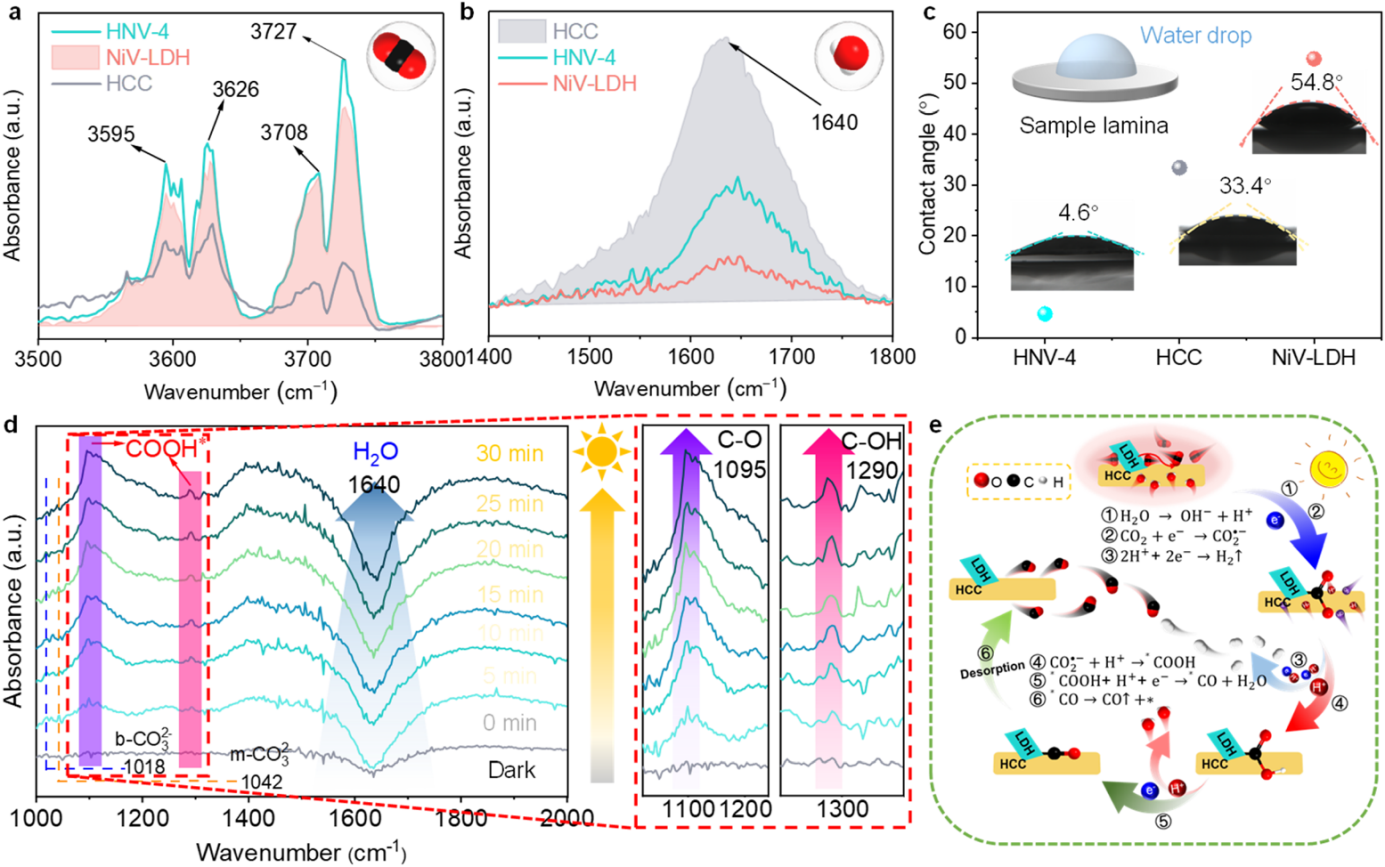
(a) DRIFTS spectra of CO_2_ adsorption for HCC, NiV-LDH, and HNV-4. (b) DRIFTS spectra of H_2_O adsorption for HCC, NiV-LDH, and HNV-4. The insets show the corresponding molecular models of adsorption (upper right corner). (c) Water contact angles of HCC, NiV-LDH, and HNV-4. The inset shows the corresponding experimental schematic (upper left corner). (d) *In situ* DRIFTS spectra for the reaction of CO_2_ with H_2_O on HNV-4 under visible light (*λ* > 420 nm). (e) Proposed reaction mechanism based on the PCET process for photocatalytic green syngas synthesis over the HNV heterojunction.

During the photoreduction of CO_2_ with H_2_O to produce syngas, the reaction microenvironment formed by the interaction between the surface of the photocatalyst and various molecules in the reaction system has an important influence on both the catalytic reaction rate and product selectivity.^58,59^ Firstly, to investigate the actual adsorption state of reactant CO_2_ molecules, we further characterize the as-prepared samples using diffuse reflectance infrared Fourier transform spectroscopy (DRIFTS).^60^ The characteristic CO_2_ absorption band with corresponding infrared peaks at 3595–3727 cm^−1^ can reflect the gas-phase CO_2_ adsorption capability. As shown in Fig. 5a, the CO_2_ adsorption ability follows the order of HNV-4 > NiV-LDH > HCC, indicating that the loading of NiV-LDH in the heterojunctions can facilitate CO_2_ enrichment on the surface of the samples. The underlying reason is probably due to the intrinsic alkaline property of the LDH that makes it easier to capture acidic CO_2_ molecules.^26^ When NiV-LDH is assembled with HCC, the chemical interaction of HNV-4 with CO_2_ is further enhanced due to the synergistic effect between the components, which favors the reduction of the chemically activated bond energies and reaction barriers of O=C=O. Compared with that for NiV-LDH, the DRIFTS spectrum for HCC shows the highest absorption peak at 1640 cm^−1^ (Fig. 5b), which is attributed to the vibration of adsorbed water molecules on the surface of the materials.^61^ From the above results, the adsorption capability of the samples for CO_2_ and H_2_O molecules is in good agreement with the distribution of their catalytic reduction products, where both H_2_ and CO are contained in the products of HCC with high H_2_ yields, and no H_2_ is produced by NiV-LDH, with the products containing only CO and trace CH_4_. After introducing the NiV-LDH NSs, the absorption peak strength of H_2_O molecules decreased for HNV-4 compared with HCC, suggesting that the NiV-LDH NSs could efficiently modulate the reaction microenvironment of the HNV-4 heterojunction. Additionally, similar variations are exhibited in the water contact angle (WCA) results of Fig. 5c. The WCA from small to large is HNV-4 < HCC < NiV-LDH, and a smaller contact angle can lead to adsorption of more water molecules, which is favorable for H^+^ production.^10^

The pathway of the reduction of CO_2_ to CO over HNV-4 has been investigated by *in situ* DRIFTS. Upon visible light irradiation, the absorption peaks at 1018 and 1042 cm^−1^ are assigned to the C–O stretching of the bidentate carbonate (b-CO_3_^2−^) and the monodentate carbonate (m-CO_3_^2−^) group, respectively, as a result of dissolved CO_2_ in water (Fig. 5d).^21^ The signals of the adsorbed carbonate species over NiV-LDH are similar to those observed over NHV-4 and obvious as compared with HCC (Fig. S11†). The abundant alkaline hydroxyl group on LDH should be responsible for the favorable CO_2_ adsorption process,^37^ as reflected by the results in Fig. 5a. Noteworthy, the negative peak located at *ca.* 1640 cm^−1^ belongs to the H_2_O molecules adsorbed on the surface of the sample, and the peak intensity increases gradually with the increase of the irradiation time, indicating that the water molecules adsorbed on the HNV-4 surface are continuously dissociated as the reaction proceeds. Then, CO_2_ molecules adsorbed on the surface of the photocatalyst further react with H^+^ produced by the dissociation of H_2_O to produce *COOH intermediates. As shown in the localized zoomed-in image on the right-hand side of Fig. 5d, *COOH is the most important intermediate produced by CO_2_ activation, which gives rise to the peaks at 1095 and 1290 cm^−1^ (C–O and C–OH stretching, respectively) with increased light irradiation time, proving that the photocatalytic CO_2_ reduction process based on HNV-4 is consistent with the carbene pathway.^44^

For the CO_2_ photocatalytic reduction reaction, electron transfer is often accompanied by simultaneous proton migration in the redox reaction, which is a PCET process. It is widely accepted that the PCET process is kinetically favored over electron transfer or proton transfer, and obtaining H^+^ by dissociating H_2_O molecules, which is usually used as the H^+^ source for CO_2_ reduction in aprotic solvents.^8,35,62^ As shown in Fig. 5e, based on the synergistic effect of the optimized electric field microenvironment and the reaction microenvironment, the photoreduction process of CO_2_ to green syngas over the HNV heterojunction is as follows. Some of the H_2_O molecules adsorbed on the surface of HCC and H^+^ come from the dissociation of H_2_O molecules (eqn (1)). Meanwhile, NiV-LDH NSs capture CO_2_ molecules from the surrounding reaction liquid and induce their enrichment in the HNV heterojunction to form a high local concentration of CO_2_. Then, the adsorbed CO_2_ molecules are further activated to CO_2_˙^−^ (eqn (2)). A part of the H^+^ is reduced by photogenerated electrons produced from the S-scheme HNV heterojunction after photoexcitation to form H_2_ (eqn (3)). The activated CO_2_˙^−^ is further hydrogenated with the two-step PCET process to form the vital intermediates of *COOH and *CO (eqn (4) and (5)).^63^ Finally is *CO desorption and spillover from the photocatalyst surface (eqn (6)). At this point, NiV-LDH NSs rationally modulate the microenvironment of the HNV heterojunction, both of the electric field microenvironment and the reaction microenvironment. In detail, NiV-LDH NSs serve as the OP to accelerate charge carrier separation based on the charge transfer pathway of the S-scheme, and while serving as CO_2_ collector to increase CO_2_ concentration around the HCC (RP) active sites. Based on the synergistic effect, the PCET process of the HNV-4 heterojunction is accelerated to promote the evolution of CO; simultaneously the production of H_2_ is also modulated, resulting in high green syngas yield and an extended modulation of CO/H_2_ ratio.

1 H_2_O → OH^−^ + H^+^

2 CO_2_ + e^−^ → CO_2_˙^−^

3 2H^+^ + 2e^−^ → H_2_↑

4 CO_2_˙^−^ + H^+^ → *COOH

5 *COOH + H^+^ + e^−^ → *CO + H_2_O

6 *CO → CO↑ + *

## 4. Conclusions

A highly efficient CdS@NiV-LDH (HNV) photocatalyst is designed and fabricated *via* a simple oil bath method, in which NiV-LDH NSs are uniformly grown *in situ* on the surface of hollow cubic CdS. The HNV heterojunction photocatalyst represents a simple but highly effective analog of photosynthesizing plants, in which the NiV-LDH NSs mainly act as CO_2_ collector and light absorber and HCC offers the catalytic sites for both H_2_O and CO_2_. The electric field microenvironment is modified by redistributing space charge *via* a built-in electric field, while the adjusted reaction microenvironment promotes the local enrichment of CO_2_ molecules, leading to the effective modulation of the PCET reaction process and facilitating the formation of *COOH intermediates over the prepared S-scheme heterojunction photocatalysts. As a result, the CO and H_2_ evolution rates over the optimal HNV-4 heterojunction are 1163.8 μmol g^−1^ h^−1^ and 1334.6 μmol g^−1^ h^−1^, respectively, with an optimized CO/H_2_ ratio of 0.87 and an overall syngas formation rate of 2498.4 μmol g^−1^ h^−1^. Moreover, adjustable CO/H_2_ ratios ranging from 0.2 to 1 are afforded by adjusting the loading of NiV-LDH NSs. This work provides a distinctive insight into the modulation of the PCET reaction process through the rational design and optimization of the local microenvironment of heterojunction photocatalysts and achieves satisfactory CO_2_ photoreduction activity and flexible regulation of green syngas ratio.

## Data availability

All data supporting the findings of this study are available within the paper and its ESI files.†

## Author contributions

Senlin Zhang analyzed all data and drafted the manuscript. Yuheng Ma performed the experiments and gathered all the data. Changqiang Yu, Zhaohui Huang, and Ruoning Zhan help to check the manuscript. Yingxinjie Wang carried out water contact angle tests. Xiuqiang Xie and Nan Zhang co-guided this work and corrected the manuscript. All authors contributed to a critical discussion of the data and manuscript.

## Conflicts of interest

There are no conflicts to declare.

## Supplementary Material

SC-OLF-D4SC07856J-s001
