# Supplementary material for: Modulating the PCET process via optimizing the local microenvironment of a CdS@NiV-LDH heterojunction for CO2 reduction in tunable green syngas photosynthesis
Source: Chem Sci. 2025 Feb 10;16(12):5241–51. doi: 10.1039/d4sc07856j (PMC11844268; doi:10.1039/d4sc07856j)
Supplement: SC-016-D4SC07856J-s001 [file SC-016-D4SC07856J-s001.pdf]

Supplementary Information (SI) for Chemical Science.
This journal is © The Royal Society of Chemistry 2025

Modulating PCET Process *via* Optimizing Local Microenvironment of CdS@NiV-LDH Heterojunction for CO_2 Reduction to Tunable Green Syngas Photosynthesis

Senlin Zhang,[a‡] Yuheng Ma,[a‡] Changqiang Yu,[a] Zhaohui Huang,[a] Ruoning Zhan,[a] Yingxinjie Wang,[a] Xiuqiang Xie[*a] and Nan Zhang[*a]

[a] College of Materials Science and Engineering, Hunan University, Changsha 410082, China. E-mail: xiuqiang_xie@hnu.edu.cn; nanzhang@hnu.edu.cn

Table of Contents

Experimental section S1

Fig. S1 The X-ray diffraction (XRD) patterns of HCC, NiV-LDH, and HNV-X (X = 1, 2, 3, 4, 5). S5

Fig. S2 SEM images of (a) HCC, (b) NiV-LDH, and (c) HNV-4. ....S7

Fig. S3 The image intensity line profiles of (a) HCC and (b) NiV-LDH. S7

Fig. S4 XPS survey spectra of HCC, NiV-LDH, and HNV-4. ....S8

Fig. S5 The Tauc plots of (a) HCC and (b) NiV-LDH. S9

Fig. S6 UV-vis diffuse reflectance spectra (DRS) of HCC, NiV-LDH, HNV-X (X=1, 2, 3, 4, 5). S10

Fig. S7 AQY results and corresponding UV-vis DRS for HNV-4. ....S11

Fig. S8 (a) XPS survey spectra of HNV-4 before and after reaction. High-resolution XPS spectra of (b) Cd 3d, (c) S 2p (d) Ni 2p, (e) V 2p, and (f) O 1s of HNV-4 before and after reaction. S12

Fig. S9 The image intensity line profiles of (a) HCC and (b) NiV LDH, (c) Pt nanoparticles. S13

Fig. S10 Electrochemical impedance spectroscopy (EIS) of HCC, NiV-LDH, and HNV-4. ....S14

Fig. S11 *In-situ* DRIFTS spectra for the reaction of CO_2 with H_2O on pure (a) NiV-LDH and (b) HCC under visible light ($\lambda > 420$ nm). S15

Table S1 The content of each component in the preparation process for the HNV composites. S2

Table S2 The mass percentage of Cd element for HNV-4 from the ICP-OES result. S6

Table S3 The relevant parameters for AQY calculations of HNV-4. ....S11

Table S4 Comparison of the photocatalytic CO_2 reduction performance for CdS-based and LDH-based photocatalysts in this work and previous literature. S16

References S18

Experimental section

Materials

Tris(2, 2'-bipyridine)ruthenium(II) chloride hexahydrate ($C_{30}H_{26}ClN_6ORu^+$, 99.95%, $[Ru(bpy)_3]Cl_2·6H_2O$) was purchased from Sigma-Aladdin. Polyvinylpyrrolidone ($(C_6H_9NO)_n$, *M*w = 1.3 × 10^6, PVP) and sodium sulfide (Na_2S, 95%) were obtained from Sahn Chemical Technology (Shanghai) Co., Ltd. (China). Potassium hexacyanocobaltate(III) ($K_3[Co(CN)_6]$, 98%) and Vanadium trichloride (VCl_3, 96%) were obtained from Aladdin Biochemical Reagent Co., Ltd. (Shanghai, China). Cadmium chloride hemipentahydrate ($CdCl_2·2.5H_2O$, 98%), sodium citrate ($C_6H_5Na_3O_7·2H_2O$, 99%), ammonium hydroxide ($NH_3·H_2O$, 25-28 wt.%), nickel(II) nitrate hexahydrate ($Ni(NO_3)_2·6H_2O$, ≥ 98%), Hexamethylene tetramine ($C_6H_{12}N_4$, ≥ 98%, HMTA), thioacetamide (C_2H_5NS, ≥ 99%, TAA), anhydrous ethanol (C_2H_6O, ≥ 99.7%), Acetonitrile (CH_3CN, ≥ 99.8%), and Triethanolamine ($C_6H_{15}NO_3$, AR, TEOA) were obtained from Sinopharm Chemical Reagent Co., Ltd. (China). All materials were used as received without further treatment. Deionized water (DI, 18.2 mΩ·cm resistivity) is used throughout the experiments.

Synthesis of Cd PBA Precursor

The Cd PBA (Prussian blue analog) precursor was prepared under mild conditions. Specifically, 3.0 mmol of $CdCl_2·2.5H_2O$, 5.00 g of PVP, and 1.75 mmol of $C_6H_5Na_3O_7·2H_2O$ were dissolved in 100 mL of DI to form solution A. Then, solution B was prepared by dissolving 2.0 mmol of $K_3[Co(CN)_6]$ in another 100 mL of DI, which was added to solution A. After aging for 1 h, the obtained products were collected and washed with ethanol five times, which was then dried in an oven at 60 °C overnight.

Synthesis of CdS Hollow Cubes

A hollow CdS cube (HCC) was obtained by the sulfidation process at room temperature. The prepared Cd PBA (100 mg) was dissolved in 100 mL of mixed solution with DI and ethanol (v:v = 1:1). Then, 400 mg of TAA was dissolved. When the solution was well stirred, 800 μL of $NH_3·H_2O$ was added to the solution with gentle stirring. After reaction 1 h, 1 mL of 100 mM Na_2S solution was added. After the reaction for 30 min, the product was collected by centrifugation and washed with ethanol three times, which was then dried in an oven at 60 °C overnight.

Synthesis of NiV-layered double hydroxide (NiV-LDH)

NiV-LDH nanosheets were obtained by the oil bath method. During the preparation process, 1.0 mmol

of $Ni(NO_3)_2{\cdot}6H_2O$, 2.0 mmol of VCl_3, 2.0 g of PVP, and 50 mL of DI are added into a 100 mL of round-bottom flask. After stirring for 10 min, 3.0 mmol of HMTA and 0.3 mmol of $C_6H_5Na_3O_7{\cdot}2H_2O$ were added into the above solution with vigorous stirring. After stirring for 30 min, put the round-bottom flask in the oil bath with a reflux condenser, and maintain it at 120 °C for 12 h. Finally, the product was collected by centrifugation and washed with ethanol three times, which was then dried in an oven at 60 °C for 12 h.

Synthesis of Cd@NiV-LDH (HNV)

HNV was obtained by the *in-situ* one-pot oil bath method. Specifically, 20 mg of HCC and 50 mL of DI are added into a 100 mL round-bottom flask. After ultrasonic treatment for 5 min, the HCC was uniformly dispersed in aqueous solution. Then, 10 μmol of $Ni(NO_3)_2{\cdot}6H_2O$, 20 μmol of VCl_3, and 200 mg of PVP into the above solution, respectively. After stirring for 10 min, 2.5 mmol of HMTA and 2.5 mmol of $C_6H_5Na_3O_7{\cdot}2H_2O$ were added into the above solution with continuous stirring. When the pH of the solution turned to about 10, the round-bottom flask was in the oil bath with a reflux condenser and maintained at 120 °C for 12 h. The precipitate was collected by centrifugation and washed with ethanol three times, which was then dried in an oven at 60 °C for 12 h. The load of NiV-LDH on HCC (HNV-X, X = 1, 2, 3, 4, 5) could be regulated by controlling the mass of the $Ni(NO_3)_2{\cdot}6H_2O$ and VCl_3 (Table S1†).

Table S1 The content of each component in the preparation process for the HNV composites.

Sample	CdS (mg)	$Ni(NO_3)_2{\cdot}6H_2O$ (μmol)	VCl_3 (μmol)
**HNV-1**	20	10	20
**HNV-2**	20	20	40
**HNV-3**	20	40	80
**HNV-4**	20	60	120
**HNV-5**	20	80	160

Characterization

A miniFlex diffractometer (Rigaku, Japan) with Ni-filtered Cu Kα radiation is employed to measure X-ray diffraction (XRD) patterns. Geometrical morphology is observed by S-4800 scanning electron microscope (SEM, Hitachi, Japan). Transmission electron microscopy (TEM) and high-resolution TEM (HR-TEM) images were acquired on a JEOL JEM-2100F microscope with a 200 kV accelerating voltage. A Thermo Scientific AXIS SUPRA spectrometer (Thermo Fisher Scientific, USA) is utilized to gather

X-ray photoelectron spectra (XPS) signal, which was made of a monochromatic Al Kα as the X-ray source, and all of the binding energies were calibrated by the C 1s peak at 284.8 eV. A UV-2600 spectrophotometer (Shimadzu, Japan) is utilized to measure the diffuse reflectance spectra (DRS) of the powder samples using $BaSO_4$ as reference material. Electron spin resonance (ESR) was performed on an ESR spectrometer (MEX-nano, Bruker, DEU). Water contact angles were tested by contact angle measuring device (SINDIN, SDC-100) at room temperature. *In-situ* diffuse reflectance infrared Fourier transform spectroscopy (DRIFTS) was obtained on a Nicolet iS50 FTIR spectrometer (Thermo Fisher, USA) equipped with a mercury cadmium telluride detector. CO_2 and H_2O adsorption experiments were performed by testing the diffuse reflectance infrared Fourier transform spectroscopy of the samples after CO_2-containing water vapor was introduced for 5 min. The isotope labeling experiments were performed using $^{13}CO_2$ instead of $^{12}CO_2$, and the products were analyzed using gas chromatograph-mass spectrometry (GC-MS, 7890A and 5975C, Agilent). The element content is quantitatively analyzed by inductively coupled plasma optical emission spectrometer (ICP-OES, Agilent 720, Agilent).

Photoelectrochemical measurements

The assessment of photoelectrochemical properties is conducted based on a CHI 660E electrochemical station (Shanghai Chenhua, China). The whole experiments are carried out within a Pyrex-glass cell using Ag/AgCl and Pt plate (10 mm × 10 mm) as reference electrode and counter electrode, respectively. The work electrode is prepared through the following steps. First, the fluorine-doped tin oxide (FTO) glass is thoroughly washed with ethanol by sonication. Second, after drying, the FTO glass is attached with Scotch tape that has a circular hole with an area of 0.25 cm^2. Third, 5 mg of the samples were dispersed in 500 μL of N, N-dimethylformamide (DMF) assisted by an ultrasonic concussion to form a quasi-steady suspension, which was then spread onto the hole left on the pretreated FTO glass. Finally, after the suspension is naturally dried, the affixed scotch tape is carefully dislodged from the glass, and the other region that is not covered with the sample is insulated by epoxy glue. Photocurrent curves are measured in 0.2 M Na_2SO_4 aqueous solution at the open-circuit potential under the irradiation by a 300 W Xe lamp (PLS-SXE300+, Beijing Perfect Light Co., Ltd.) equipped with a 420 nm cut-off filter. The electrochemical impedance spectroscopy (EIS) experiments were conducted with the electrolyte of 0.5 M KCl aqueous solution containing 10 mM $K_3[Fe(CN)_6]/K_4[Fe(CN)_6]$ (1:1) under open-circuit potential conditions. The Mott-Schottky (M-S) experiments were measured in 0.2 M Na_2SO_4 aqueous solution.

Measurement of Photocatalytic CO_2 Reduction

Reactions of photocatalytic CO_2 reduction were conducted in a custom-made reactor with a volume of 35 mL, and a 300 W Xe lamp with a 420 nm cutoff filter (PLS-SXE 300E, Beijing Perfect Light Co. Ltd.)

was used as the light source. Specifically, 2.0 mg of photocatalyst and 6.0 mg of $[Ru(bpy)_3]Cl_2 \cdot 6H_2O$ were added to a solution of DI (2.0 mL), CH_3CN (6 mL), and TEOA (2.0 mL) and dispersed with the help of sonication. After degassing and backfilling the mixture with CO_2 (99.999%) for 30 min, the mixture was then irradiated under stirring for photocatalytic CO_2 reduction. The temperature of the photocatalytic system is maintained at 25 °C by cooling cycling water. After every 1 h of light irradiation, 1 mL of the gas product was sampled with a gastight syringe and analyzed using a gas chromatograph (Shimadzu GC-2014, 5 A molecular sieve column). The H_2 was detected by the TCD detector, and the CO and CH_4 were detected by the FID1 detector. Control experiments were conducted in the absence of light irradiation, a photocatalyst, a sacrificial reagent, and CO_2. The recycling experiments for photocatalytic CO_2 reduction were performed under the same conditions to evaluate the stability of the catalyst in a long-term operation. After each photocatalytic activity test, the used photocatalyst was separated, washed with ethanol, and then dried at 60 °C in the vacuum oven for the next test.

Calculation of Apparent Quantum Yield of Syngas Production

The AQY value is calculated based on the following equations:

$$N = \frac{I \times A \times t \times \lambda}{h \times c}$$

$$AQY = \frac{2 \times \text{the number of evolved syngas molecules}}{N} \times 100\%$$

Where I is the measured light intensity of monochromatic light, A is the illumination area (3.92×10^{-4} m^2), t is the illumination time (3600 s), h is the Plank constant (6.62×10^{-34} J·s), c is the light speed (3×10^8 m/s), and λ is the monochromatic light's wavelength, and 2 represents the number of consumed electrons of CO_2 photoreduction for the formation of syngas.

Experiment of *in-situ* photodeposition

For HNV-4@Pt, 4 mg of HNV-4 photocatalyst powder was added to 12 mL of a 9:1 DI/methanol mixture and dispersed by sonication. Then, $H_2PtCl_6 \cdot H_2O$ solution containing 3 wt.% Pt relative to the photocatalyst was added dropwise to the above suspension. After degassing and backfilling the mixture with N_2 for 30 min under darkness, the suspension was then irradiated for 1 hour with a 300 W Xe lamp ($\lambda > 420$ nm) while being continuously stirred and N_2 flow. After photo-deposition, the suspension was washed with DI two times and freeze-dried for the subsequent characterization.

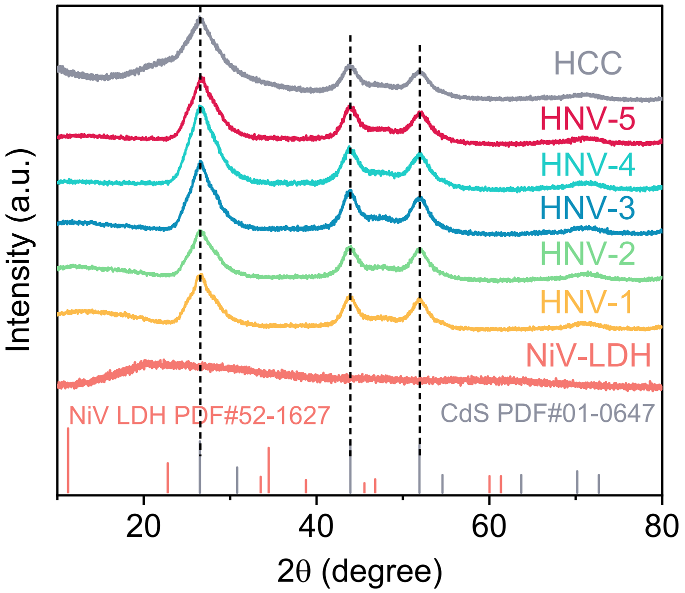

Fig. S1 The X-ray diffraction (XRD) patterns of HCC, NiV-LDH, and HNV-X (X = 1, 2, 3, 4, 5).

Supplementary Note: With increasing NiV-LDH nanosheets (NSs) loading, there is no significant change in the characteristic peaks of HCC and the diffraction pattern of NiV-LDH cannot be observed clearly in HNV heterostructure, which is caused by introducing a low content of NiV-LDH NSs in the composite.[1]

Table S2 The mass percentage of Cd element for HNV-4 from the ICP-OES result.

M (mg)[a]	V (mL)[b]	Element	wt. (%)[c]
385	25	Cd	40.47

[a] M is the mass of the sample. [b] V is the constant volume of the measurement. [c] wt. is the mass percent of the element.

Supplementary Note: The layered double hydroxides (LDHs) are represented by the general formula $M^{II}_{1-x}M^{III}_{x}(OH)_2(A^{n-})_{x/n}\cdot\gamma H_2O$, where M^{II} and M^{III} refer to metallic divalent (Ni^{2+}) and trivalent cations (V^{3+}), respectively, and A^{n-} denotes the CO_3^{2-} for the NiV-LDH. Considering the fact that NiV-LDH contains intercalated anion CO_3^{2-} and interlayer water molecules,[2] the accurate content of NiV-LDH in the HNV composite cannot be determined through measuring the metal elements (i.e., Ni and V) contents. Therefore, the content of CdS is analyzed and then the ratio of NiV-LDH in the composite is calculated. The result indicates that the NiV-LDH nanosheets loading content in HNV-4 is 48.0 wt.%.

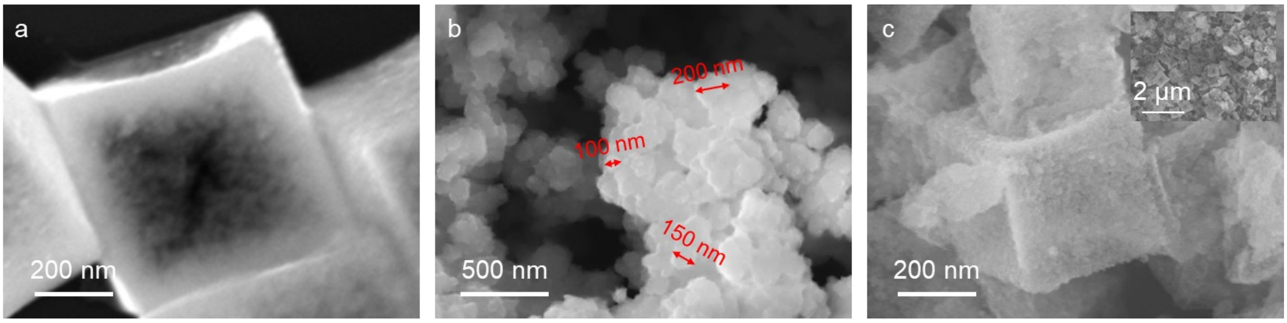

Fig. S2 SEM images of (a) HCC, (b) NiV-LDH, and (c) HNV-4.

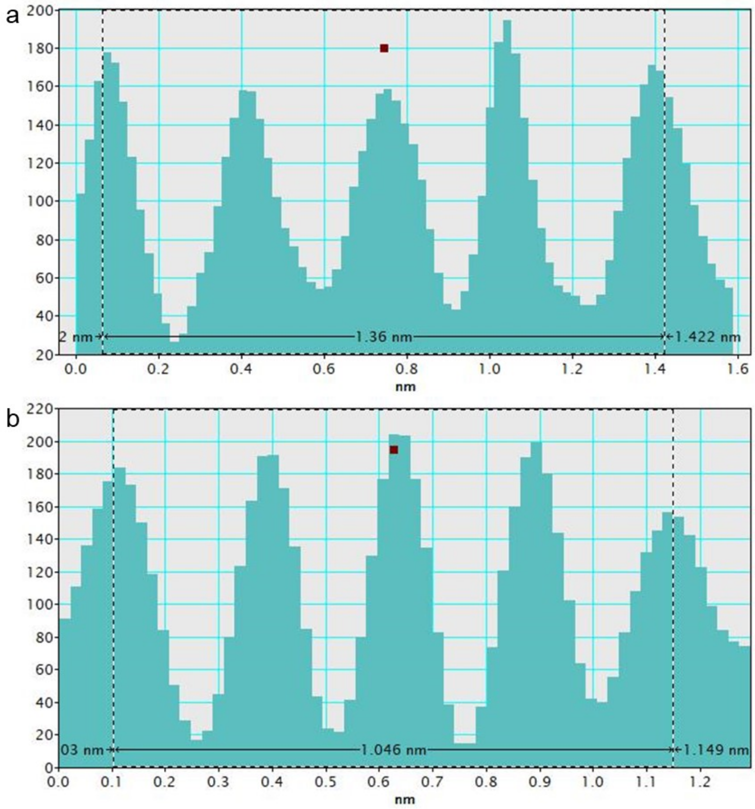

Fig. S3 The image intensity line profiles of (a) HCC and (b) NiV-LDH.

Supplementary Note: Calculated from the image intensity line profiles, the lattice fringe spacing is 3.40 Å and 2.61 Å, corresponding to the (111) and (012) crystal facets of cubic CdS and NiV-LDH, respectively.[3]

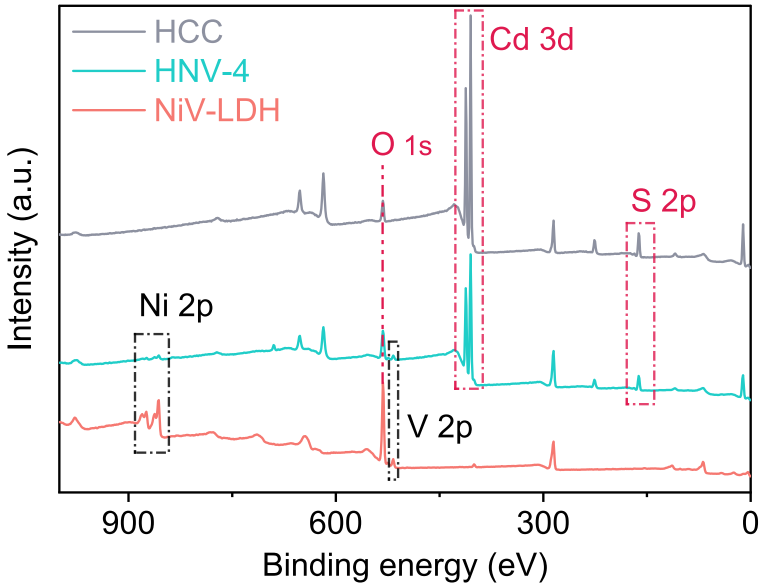

Fig. S4 XPS survey spectra of HCC, NiV-LDH, and HNV-4.

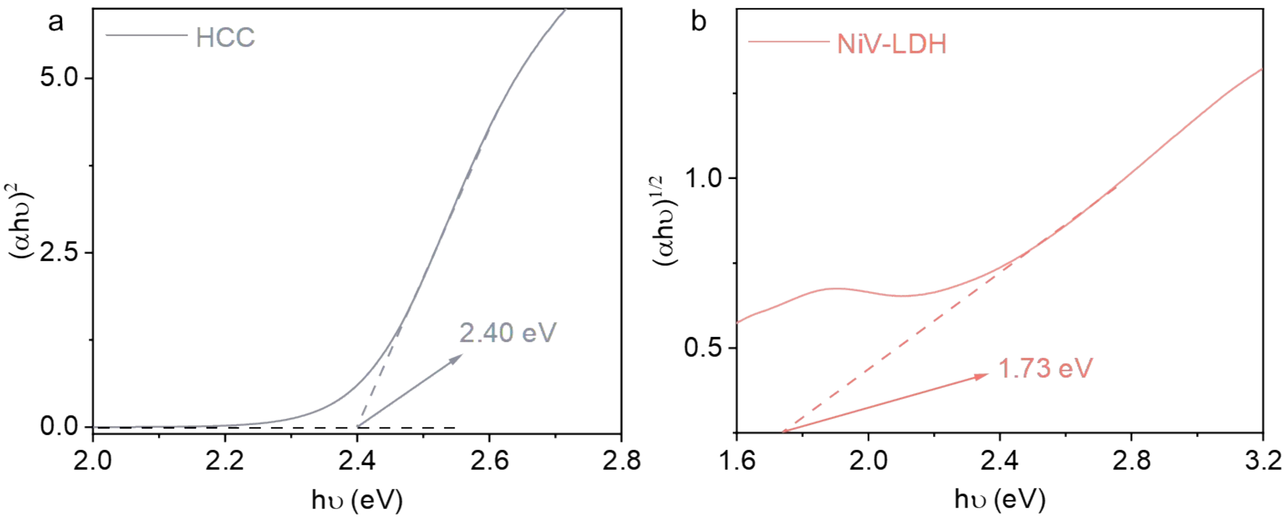

Fig. S5 The Tauc plots of (a) HCC and (b) NiV-LDH.

Supplementary Note: The bandgaps of HCC and NiV-LDH are calculated by Tauc's equation: $(\alpha h\upsilon)^{1/n} = A(h\upsilon - E_g)$, in which α, *h*, υ, A, and E_g represent the absorption coefficient, Plank constant, light frequency, proportionality, and bandgap energy, respectively.[4] Besides, the value of n depends on the transition characteristics of semiconductors, which is 1/2 for CdS (a direct-gap semiconductor) while 2 for NiV-LDH (an indirect-gap semiconductor).[5,6] As shown in Fig. S5†, the estimated E_g for HCC and NiV-LDH are 2.40 and 1.73 eV, respectively.

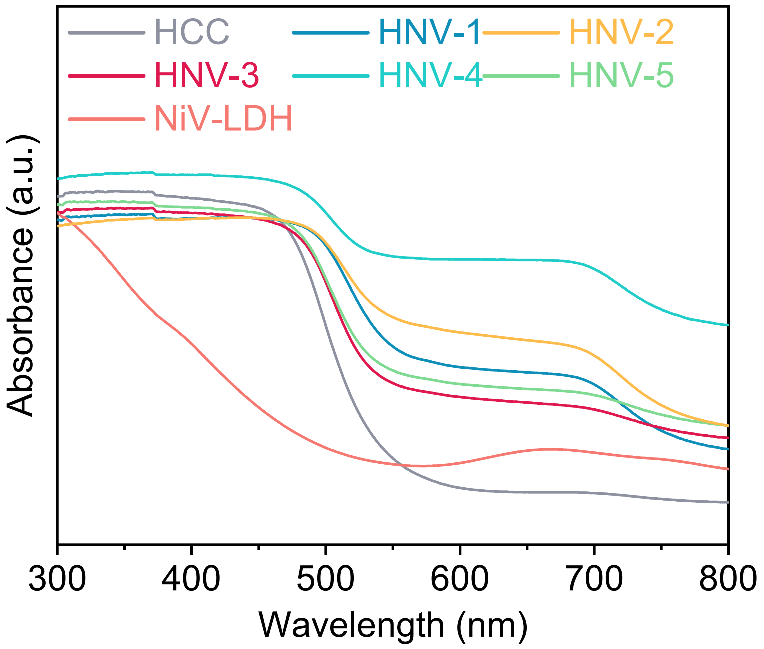

Fig. S6 UV-vis DRS of HCC, NiV-LDH, HNV-X (X=1, 2, 3, 4, 5).

Supplementary Note: Compared with the pure component, the photo-harvesting ability at above 500 nm is improved to different intensities with increasing NiV-LDH contents of HNV composites.[7] In addition, some distinctions are present in the absorption intensities at both UV and visible light regions for the different HNV-X (X=1, 2, 3, 4, 5).

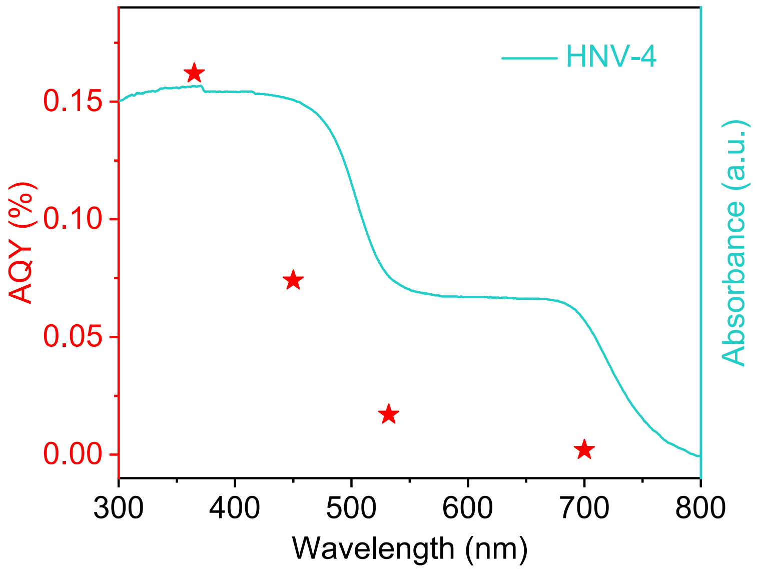

Fig. S7 AQY results and corresponding UV-vis DRS for HNV-4.

Table S3 The relevant parameters for AQY calculations of HNV-4.

**Photosensitizer**	**Monochromatic light wavelength (nm)**	**Number of incident photons**	**Amount of syngas (μmol)**	**Number of electrons consumed**	**AQY (%)**
$[Ru(bpy)_3]Cl_2·6H_2O$	365	$1.56×10^{20}$	0.24	$2.87×10^{17}$	0.184
	450	$9.92×10^{20}$	4.99	$6.01×10^{18}$	0.606
	500	$1.42×10^{21}$	0.29	$3.51×10^{17}$	0.025
	605	$2.28×10^{21}$	0.064	$7.74×10^{16}$	0.003
N/A	365	$1.56×10^{20}$	0.210	$2.52×10^{17}$	0.162
	450	$9.92×10^{20}$	0.611	$7.36×10^{17}$	0.074
	532	$1.59×10^{21}$	0.219	$2.64×10^{17}$	0.017
	700	$6.12×10^{21}$	0.080	$9.57×10^{16}$	0.002

Supplementary Note: The trend of the AQY with different incident light wavelengths matches with the UV-vis DRS of HNV-4, which further indicates that HNV-4 can effectively capture photons and convert photons into photogenerated electrons to drive the photoreduction of CO_2.

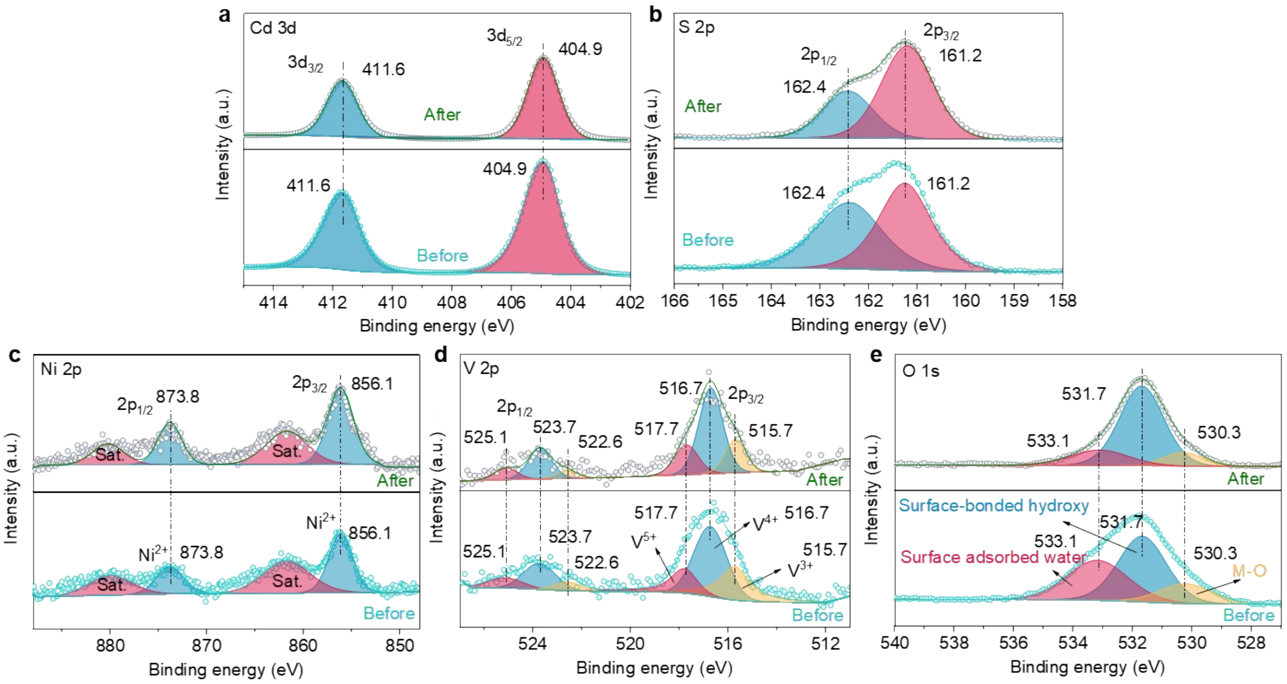

Fig. S8 High-resolution XPS spectra of (a) Cd 3d, (b) S 2p (c) Ni 2p, (d) V 2p, and (e) O 1s of HNV-4 before and after reaction.

Supplementary Note: Compared with the pristine one, the elemental composition and chemical states of the samples collected after the photocatalytic reaction show almost no obvious changes, indicating that the as-prepared HNV-4 possesses favorable photostability. Moreover, the O 1s XPS peak at 533.1 eV of the used photocatalyst is weaker than that of the pristine one. This may be due to the decomposition of adsorbed water on the surface of the HNV-4 photocatalyst during the photocatalytic production of green syngas.[8]

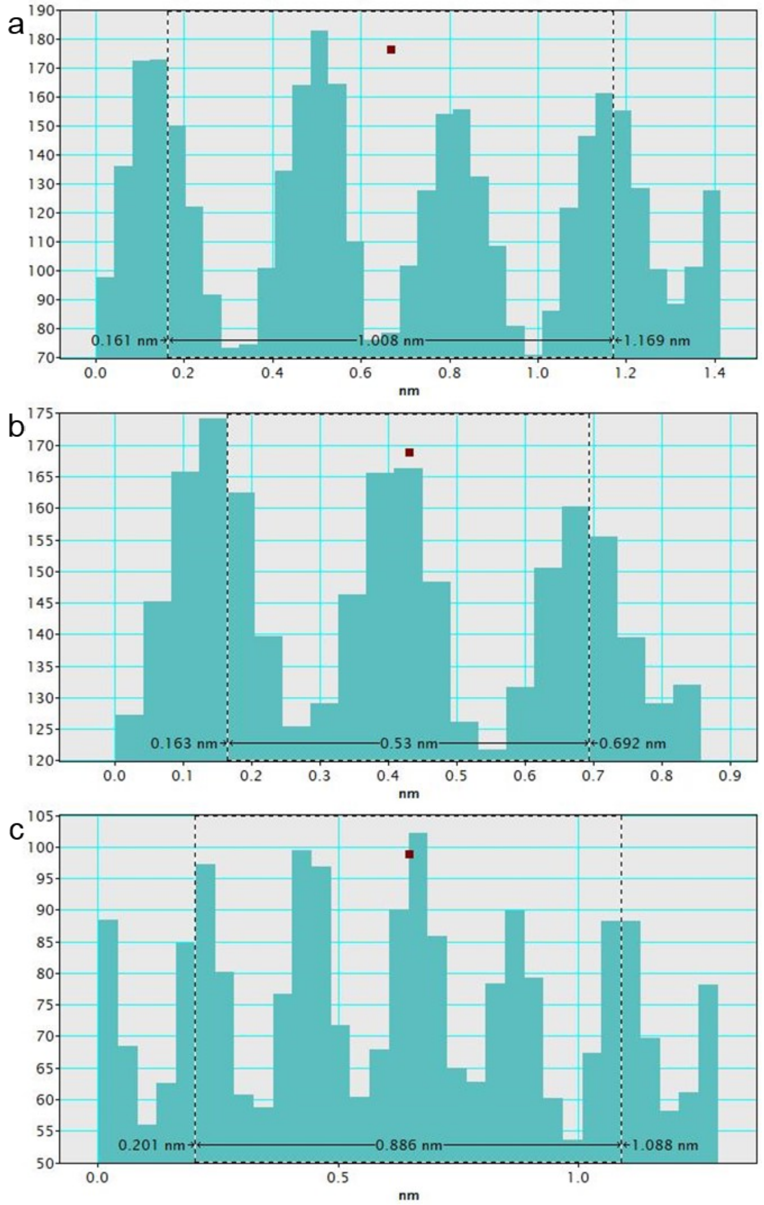

Fig. S9 The image intensity line profiles of (a) HCC and (b) NiV LDH, (c) Pt nanoparticles.

Supplementary Note: Calculated from the image intensity line profiles, the lattice fringes of ca. 3.36, 2.65, and 2.22 Å, matching well with the (111), (012), and (111) crystal facets of HCC, NiV-LDH, and Pt nanoparticles (Pt NPs), respectively.[9]

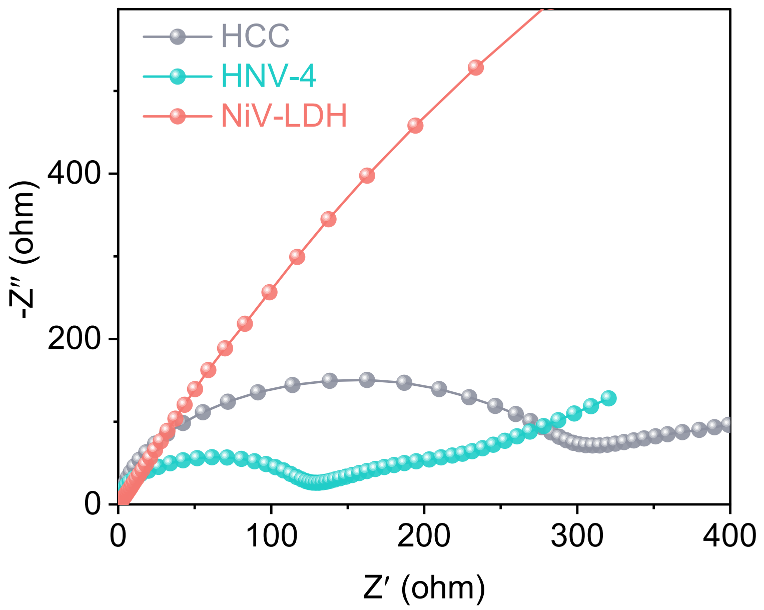

Fig. S10 Electrochemical impedance spectroscopy (EIS) of HCC, NiV-LDH, and HNV-4.

Supplementary Note: The EIS shows that HNV-4 owns a smaller high-frequency semicircle in the Nyquist plot than its two individual components, reflecting that the heterostructure possesses the lower electronic resistance, which is beneficial for accelerating separation and transfer of charge carriers in photocatalysis.[10]

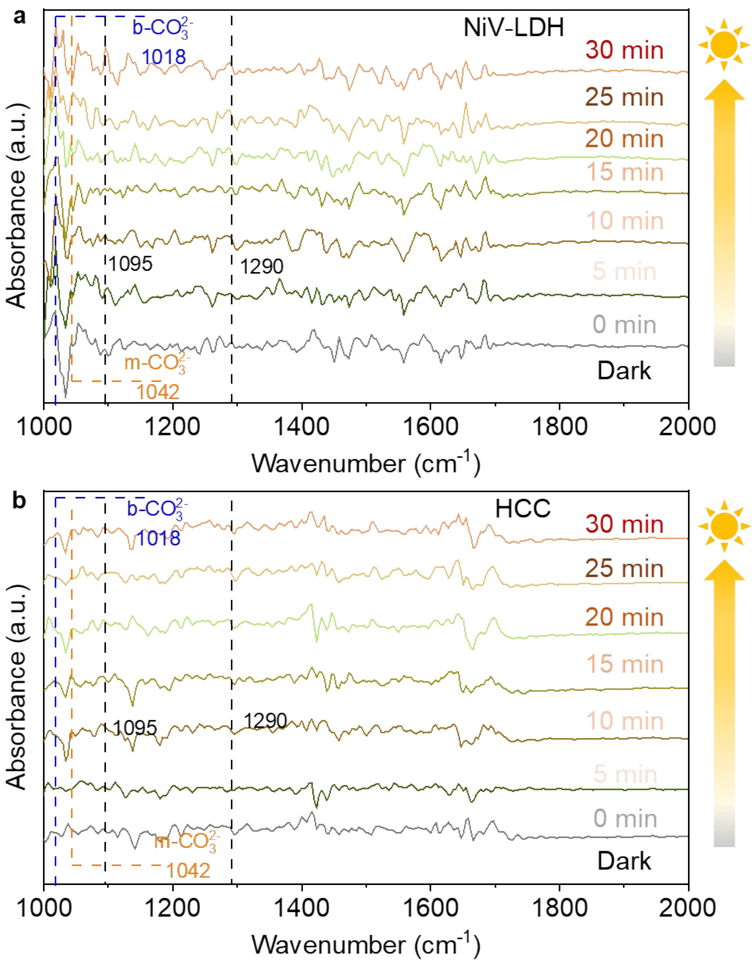

Fig. S11 *In-situ* DRIFTS spectra for the reaction of CO_2 with H_2O on pure (a) NiV-LDH and (b) HCC under visible light (λ > 420 nm).

Supplementary Note: For NiV-LDH, after dark adsorption of CO_2 with water for 10 min and then continued light irradiation, it shows strong absorption signals for both bidentate carbonate (b-CO_3^{2-}) and monodentate carbonate (m-CO_3^{2-}) groups, which indicate that NiV-LDH can adsorb CO_2 efficiently.[11] However, with the prolongation of light irradiation, its carbonate absorption peaks do not weaken obviously, and there are no obvious absorption peaks of *COOH intermediate radicals. These results indicate that NiV-LDH alone cannot effectively activate CO_2 and reduce it to CO. For HCC, the absorption intensity of carbonate groups is much weaker than that of NiV-LDH, and there is no signal of *COOH intermediate radicals detected. These results indicate that both NiV-LDH and HCC alone cannot achieve the effective photoreduction of CO_2 with water to syngas. After combining NiV-LDH nanosheets in-situ grown on the surface of HCC to form heterojunction, the obtained CdS@NiV-LDH composites are capable to effectively catalyze the CO_2 and water to syngas with a wide CO/H_2 ratio range.

Table S4 Comparison of the photocatalytic CO_2 reduction performance for CdS-based and LDH-based photocatalysts in this work and previous literature.

Photocatalyst	Light source	Photosensitizer	Sacrificial agents	Evolution rate of products (μmol/g/h)	Rang of CO/H_2 ratio	AQY_{syngas} (%)	Ref.
C@P1/A1	300 W Xe lamp, λ > 420 nm	N/A	TEOA	CO: 52.6, H_2: 270.5<br>Syngas: 323.1	0.016~0.47	no mention	12
V_S-R-CdS/EDA NW	300 W Xe lamp, λ > 420 nm	N/A	TEOA	CO: 115.6, H_2: 959.4<br>Syngas: 1075.0	0.046~0.12	no mention	13
3 wt.% Ni_2P/CdS	300 W Xe lamp, λ > 420 nm	N/A	Na_2S and Na_2SO_3	CO: 50.6, H_2: 115.0<br>Syngas: 165.6	0.25~0.50	0.79 (475 nm)	14
CdS-$Ni_{(4:1)}$	LED lamp, λ = 420 nm	N/A	TEOA	CO: 2674.0, H_2: 3235.0<br>Syngas: 5909.0	0.25~3	no mention	15
CdS HMCHP	300 W Xe lamp, λ > 400 nm	$Co(bpy)_3^{2+}$	TEOA	CO: 1337.0, H_2: 1337.0<br>Syngas: 2674.0	N/A	no mention	16
CdS-CuS-2	300 W Xe lamp, λ > 400 nm	N/A	TEOA	CO: 203.4, H_2: 2416.5<br>Syngas: 2619.9	0.03~1.00	no mention	17
Ni@CdS⊂Zn/Co-ZIF-1:3	300 W Xe lamp, λ > 420 nm	$Co(bpy)_3^{2+}$	TEOA	CO: 948.5, H_2: 2070.8<br>Syngas: 3019.3	0.36~0.45	no mention	18
CoAl-LDH/MoS_2-0.2	300 W Xe lamp, λ > 400 nm	$[Ru(bpy)_3]Cl_2 \cdot 6H_2O$	TEOA	CO: 1677.0, H_2: 6483.0<br>Syngas: 8160.0	0.11~1.00	6.45 (500 nm)	19
CoMgAl-LDH	300 W Xe lamp, λ > 400 nm	$[Ru(bpy)_3]Cl_2 \cdot 6H_2O$	TEOA	CO: 956.6, H_2: 1021.1<br>Syngas: 1977.7	0.89~1.32	3.192 (405 nm)	20
$CdIn_2S_4$/PDDA/NiFe-LDH	300 W Xe lamp, λ > 420 nm	N/A	TEOA	CO: 12.4, H_2: 9.5<br>Syngas: 21.9	1.08~1.37	no mention	21
NiAl-LDH-CoPd	300 W Xe lamp, λ > 420 nm	$[Ru(bpy)_3]Cl_2 \cdot 6H_2O$	TEOA	CO: 570.7, H_2: 563.1<br>Syngas: 1133.8	0.61~1.01	0.199 (475 nm)	22
Pd/CoAl-LDH-7.57	300 W Xe lamp, λ > 400 nm	$[Ru(bpy)_3]Cl_2 \cdot 6H_2O$	TEOA	CO: 581.8, H_2: 1299.1<br>Syngas: 1880.9	0.45~1.35	no mention	23
Ce-0.15	300 W Xe lamp, λ > 400 nm	$[Ru(bpy)_3]Cl_2 \cdot 6H_2O$	TEOA	CO: 85, H_2: 110.5<br>Syngas: 195.5	0.13~0.77	1.45 (470 nm)	24

CoMgAl-LDH/TiC-1.7	300 W Xe lamp, λ > 400 nm	$[Ru(bpy)_3]Cl_2 \cdot 6H_2O$	TEOA	CO: 741.07, H_2: 1084.64<br>Syngas: 1825.7	0.25~1	no mention	25
HNV-4	300 W Xe lamp, λ > 420 nm	$Ru(bpy)_3Cl_2 \cdot 6H_2O$	TEOA	CO: 1163.9, H_2: 1334.6<br>Syngas: 2498.5	0.20~1.00	0.606 (450 nm)	This work

References

1. M. X. Yang, K. Wang, Y. B. Li, K. C. Yang and Z. L. Jin, *Appl. Surf. Sci.*, 2021, **548**, 149212.
2. C. Ning, S. Bai, J. Wang, Z. Li, Z. Han, Y. Zhao, D. O'Hare and Y.-F. Song, *Coord. Chem. Rev.*, 2023, **480**, 215008.
3. X. L. Cao, L. G. Zhang, C. Y. Guo, T. X. Chen, C. Feng, Z. C. Liu, Y. Qi, W. Wang and J. D. Wang, *Int. J. Hydrogen Energy*, 2022, **47**, 3752-3761.
4. J. H. Hua, S. Feng, C. C. Ma, H. T. Huang, K. Wei, X. J. Dai, K. D. Wu, H. H. Wang and Z. W. Bian, *J. Environ. Chem. Eng.*, 2023, **11**, 111290.
5. Y. H. Ma, Y. Zhang, G. S. Xie, Z. H. Huang, L. F. Peng, C. Q. Yu, X. Q. Xie, S. L. Qu and N. Zhang, *ACS Catal.*, 2024, **14**, 1468-1479.
6. M. Li, J. Z. Wang and Z. L. Jin, *Energy & Fuels*, 2023, **37**, 5399-5411.
7. Z. Li, Z. Liu, Y. Li and Q. Wang, *J. Alloys Compd.*, 2021, **881**, 160650.
8. M. Chi, X. Sun, G. Lozano-Blanco and B. J. Tatarchuk, *Appl. Surf. Sci.*, 2021, **570**, 151147.
9. L. F. Peng, C. Q. Yu, Y. H. Ma, G. S. Xie, X. Q. Xie, Z. J. Wu and N. Zhang, *Inorg. Chem. Front.*, 2022, **9**, 994-1005.
10. B. Su, M. Zheng, W. Lin, X. F. Lu, D. Y. Luan, S. B. Wang and X. W. (David) Lou, *Adv. Energy Mater.*, 2023, **13**, 2203290.
11. S. Yu, L. Tan, S. Bai, C. Ning, G. Liu, H. Wang, B. Liu, Y. Zhao and Y.-F. Song, *Small*, 2022, **18**, 2202334.
12. X. Yan, M. Yuan, Y. L. Yuan, P. Su, Q. Chen and F. X. Xiao, *Chem. Sci.*, 2024, **15**, 10625.
13. T. Tian, X. Y. Jin, N. Guo, H. Q. Li, Y. Han and Y. P. Yuan, *Appl. Catal., B,* 2022, **308**, 121227.
14. P. Yadav, S. Kumar, N. Velankanni, T. D. Kühne, S. Gosavi, R. K. M. Raghupathy, R. Bhosale, G. Held, M. Shelke and S. Ogale, *J. Phys. Energy*, 2024, **6**, 025019.
15. H. Y. Wang, W. H. Xie, D. D. Wei, R. Hu, N. Wang, K. Chang, S. L. Lei, B. Wang and R. Cao, *ChemSusChem*, 2022, **15**, e202200200.
16. P. Zhang, S. B. Wang, B. Y. Guan and X. W. (David) Lou, *Energy Environ. Sci.*, 2019, **12**, 164-168.
17. J. Y. Xu, X. J. Liu, H. N. Huang, Y. H. Xu, Z. Zhong, Y. F. Li, R. J. X. Zeng, J. Lü and R. Cao, *Inorg. Chem. Front.*, 2022, **9**, 2150-2160.
18. H. Z. Liu, X. Liu, B. Li, H. Q. Luo, J. G. Ma and P. Cheng, *ACS Appl. Mater. Interfaces*, 2022, **14**, 28123-28132.
19. C. H. Qiu, S. Bai, W. J. Cao, L. Tan, J. Y. Liu, Y. F. Zhao and Y. F. Song, *Trans. Tianjin Univ.*, 2020, **26**, 352-361.
20. C. J. Ning, Z. L. Wang, S. Bai, L. Tan, H. L. Dong, Y. Q. Xu, X. J. Hao, T. Y. Shen, J. W. Zhao, P. Zhao, Z. R. Li, Y. F. Zhao and Y. F. Song, *Chem. Eng. J.*, 2021, **412**, 128362.
21. Q. L. Mo, J. L. Li, S. R. Xu, K. Wang, X. Z. Ge, Y. Xiao, G. Wu and F. X. Xiao, *Adv. Funct. Mater.*, 2023, **33**, 2210332.
22. L. Yang, L. Li, P. F. Xia, H. Y. Li, J. J. Yang, X. H. Li, X. D. Zeng, X. D. Zhang, C. Xiao and Y. Xie, *Chem. Commun.*, 2021, **57**, 11629-11632.
23. X. Wang, Z. L. Wang, Y. Bai, L. Tan, Y. Q. Xu, X. J. Hao, J. K. Wang, A. H. Mahadi, Y. F. Zhao, L. R. Zheng and Y. F. Song, *J. Energy Chem.*, 2020, **46**, 1-7.
24. L. Tan, K. Peter, J. Ren, B. Y. Du, X. J. Hao, Y. F. Zhao and Y. F. Song, *Front. Chem. Sci. Eng.*, 2020, **15**, 99-108.
25. H. J. Wang, S. Bai, P. Zhao, L. Tan, C. J. Ning, G. H. Liu, J. K. Wang, T. Y. Shen, Y. F. Zhao and Y. F. Song, *Catal. Sci. Technol.*, 2021, **11**, 7091-7097.
